# Lamellar Schwann cells in the Pacinian corpuscle potentiate vibration perception

**DOI:** 10.1126/sciadv.adt5110

**Published:** 2025-06-11

**Authors:** Yuh-Tarng Chen, Dominica de Thomas Wagner, Alastair J. Loutit, Ali Nourizonoz, Marina Ulanova, Danai Graikou, Man-Shan Chang, Mary-Claude Croisier-Coeytaux, Stéphanie Clerc-Rosset, Jérôme Blanc, Graham Knott, Kuo-Sheng Lee, Daniel Huber

**Affiliations:** ^1^ Institute of Biomedical Sciences, Neuroscience Program of Academia Sinica, Academia Sinica, Taipei, Taiwan.; ^2^ Department of Basic Neurosciences, University of Geneva, Geneva, Switzerland.; ^3^ Ecole Polytechnique Fédérale Lausanne, Lausanne, Switzerland.

## Abstract

Pacinian corpuscles are among the most sensitive mechanoreceptors found in vertebrates, and they are tuned to vibrations in the highest perceptible frequency range (100 to 2000 Hz). One of their anatomical hallmarks is the onion-like cell layers surrounding the central axon. The innermost layers consist of ~60 densely packed lamellar Schwann cells (LSCs), whose function remains largely unknown. Using high-resolution three-dimensional electron microscopy, we found that LSCs do not form concentric rings, but complex, multilayered, and intertwining assemblies that are connected via a high density of desmosomes and gap junctions. LSCs make multiple converging contacts with the afferent axon via desmosomes. Using optogenetic manipulations of LSCs, we demonstrate not only that their activation drives reliable time-locked spiking in the axon but also that their inactivation significantly elevates the thresholds in situ and increases perceptual thresholds behaviorally. Together, these findings provide evidence that LSCs are a key element of somatosensory processing, actively potentiating mechanosensitivity in Pacinian corpuscles.

## INTRODUCTION

The somatosensory system is remarkably well adapted to extract vibratory stimuli from the environment, which arise from surrounding movement and when actively exploring surface textures and frictions. In vertebrates, vibrations are transduced into electrical impulses by different mechanoreceptor end-organs, such as Merkel cells, Ruffini, Krause, Meissner, and Pacinian corpuscles, or Lanceolate endings (*1*, *2*). Pacinian corpuscles (PCs) are uniquely sensitive to high-frequency substrate vibrations in the range of tens of nanometers, despite being typically located near the hypodermis and periosteum and thus further away from the skin surface compared to most other receptor types (*1*, *3*). To uncover the mechanism by which PCs achieve such exquisite vibration sensitivity requires not only a detailed understanding of their cellular structures and their respective interactions but also functional manipulations assessing their contribution to physiological activation.

PCs are extremely large compared to other mechanoreceptors (~2 mm in humans) and consist of a central elliptic cylindrical axon that often ends in a bulbing and bifurcating tip (*4*). This afferent axon terminal expresses high densities of mechanosensitive channels (*4*) and is tightly wrapped with lamellar Schwann cells (LSCs), also known as nonmyelinating sensory Schwann cells (*5*) or terminal Schwann cells (*4*), forming the inner core. In a transverse section, the Schwann cell lamellar layers appear to form two concentric semilunar halves separated by two clefts, which span across the inner core. Protrusions extend from the axon into the cleft region (*4*, *6*, *7*). The inner core is surrounded by three groups of concentrically arranged layers: first the intermediate layer, then the outer core layers, and finally the capsule. The intermediate layer consists of endoneurial CD34^+^ cells (*8*), whereas the outer core and capsule layers are made of perineural squamous epithelial cells (*9*).

How this ensemble of layered surrounding cells participates in mediating touch sensation remains unclear. Early experiments showed that the removal of the outermost layers (decapsulation) substantially affects PC kinetics and rapid adaptation (*3*, *10*). Previously proposed roles of the LSCs include the maintenance of the ionic or metabolic microenvironment (*3*, *11*), the mediation of rapid adaptation of afferent firing (*12*, *13*), and, most recently, active agents in initiating pain and somatosensation (*5*, *14*). The innermost LSC layers have been suggested to play a central role in mechanotransduction in Meissner corpuscles (*3*, *4*, *14*–*16*). In the avian Meissner corpuscle homolog, it has been shown that LSCs are mechanosensitive and excitable (*15*, *17*, *18*). Also, recent work demonstrated that LSCs surrounding mechanoreceptor nerve endings within the Meissner’s corpuscle and in hair follicle lanceolate endings set perceptual thresholds for touch (*5*). Specific subcellular elements such as tethers (*5*, *19*) that assist in opening mechanically gated channels such as Piezo2 or adherens junctions that help to stretch cellular membranes have also been proposed to play roles in mechanosensation, particularly in rapidly adapting mechanoreceptors (*4*).

Together, LSCs are ideally situated to potentiate mechanotransduction, but it remains unknown if they participate in the PC’s exquisite sensitivity to high-frequency vibration. Here, we combine high-resolution volumetric electron microscopy and optogenetic manipulation to directly assess the contribution of LSCs to the transduction of mechanical stimuli.

## RESULTS

### Ultrastructure of PC and LSCs

**Fig. 1.**
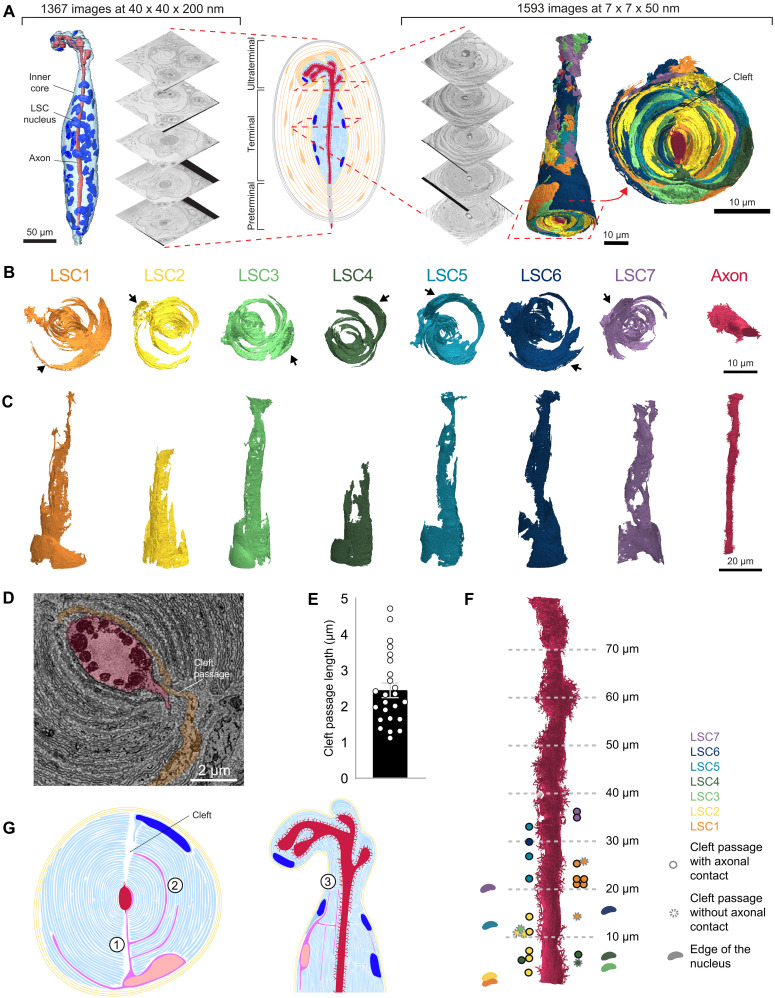
High-resolution SBF-SEM reconstructions reveal the complex morphology and surface area of the LSCs comprising the PC inner core. (**A**) Schematic showing the two image stacks acquired with SBF-SEM. The first allowed for reconstruction of all components of the PC (fig. S1). Here, we display the inner core region containing 63 LSC nuclei, with the axon in the middle. The second SBF-SEM stack is a 75-μm subsection of the inner core, which was acquired at higher resolution and allowed for the reconstruction of individual LSCs. The renderings of all seven reconstructed LSCs together show the interleaving of the layers from different cells. In the illustrated schematic, the axon is in red, the inner core is in light blue, the LSC nuclei are in dark blue, the intermediate layer is in yellow, the outer core is in orange, the capsule is in dark gray, and the myelin sheath on the axon is in light gray. (**B** and **C**) 3D renderings of the seven individual LSCs and axon reconstructed from the higher-resolution stack, seen from an *x*-*y* (B) and *x*-*z* (C) perspective. The black arrows show the position of the individual LSC nucleus. (**D**) Example cleft passage from LSC1. The cleft passage makes a contact point with an axon protrusion and leads to the formation of a new layer closer to the axon. (**E**) Length of LSC segments in the cleft. (**F**) Schematic showing LSC nuclei positions and cleft passages, including their locations and whether each cleft passage has direct axon contact. (**G**) Schematic summarizing structural characteristics of LSCs. (1) LSCs have branches that directly approach the axon from the periphery of the inner core by cutting through the cleft. (2) LSCs form many layers in the inner core. (3) LSC layers extend proximally and distally within the inner core.

To understand the detailed morphology of Pacinian LSCs, we conducted an ultrastructural analysis using serial block-face scanning electron microscopy (SBF-SEM). PCs were dissected from the hindlimb periosteum of a mouse (fig. S1) and processed for electron microscopy (see Methods). We first imaged a complete PC at “lower” resolution (voxel size: 40 nm × 40 nm × 200 nm; Fig. 1A) and manually reconstructed (see Methods) the different primary components (fig. S2). This three-dimensional (3D) reconstruction revealed what had previously been extrapolated from single-section transmission electron microscopy (TEM) and identified by conventional light microscopy (*3*, *6*, *7*, *20*, *21*), that a PC consists of tightly packed LSCs wrapping around an axon, which in turn are surrounded by the outer core consisting of multiple concentric layers of flattened cells, which are subdivided into an intermediate layer, an outer core, and a capsule (Fig. 1A and fig. S2). While the axon is a straight elliptic cylinder in the proximal portion of the PC, distinct bulbings and bifurcations are observed in the distal portion (ultraterminal) of the PC (fig. S2A).

Although at this resolution we were able to reconstruct the overall shape of the inner core, and identify the location and number of LSC nuclei (63; Fig. 1A), it was not possible to distinguish individual LSC membranes. We therefore sought to reconstruct a more detailed morphology of individual LSCs, to determine how they interact with the axon and with other LSCs. For this purpose, we processed and imaged a subsection of a PC at higher resolution (voxel: 7 nm × 7 nm × 50 nm; Fig. 1A) using SBF-SEM. The total size of the imaged block was 29 μm × 31 μm × 80 μm, encompassing the full transverse cross section of the inner core, though not its full length. We initially performed manual reconstruction of a single LSC (fig. S3A) by visual annotation. To scale up the reconstruction process and extract individual LSCs from the electron microscopy dataset, we implemented a machine learning–assisted segmentation pipeline (see Methods), using Webknossos automated segmentation services (*22*), followed by manual proofreading. A convolutional neural network (CNN) was first trained on our manual annotations and then used to generate affinity predictions, indicating the likelihood of a voxel being connected to its neighbors. Volume segments were grouped into agglomerates ranked by predicted affinity (*23*), with additional constraints—such as ensuring that each segment contained only one nucleus—to reduce merge errors. Extensive human proofreading was then performed to correct both merge and split errors, enabling accurate reconstruction of seven LSCs (Fig. 1, B and C). When viewed in the transverse plane (Fig. 1B), 3D renderings of the reconstructions reveal cells that consist of multiple lamellae that wrap around the axon in a claw-like manner, with an unevenly distributed surface area (fig. S4A), in stark contrast to previous descriptions of evenly distributed layers split by two clefts. The elongation of the cells along the axon is demonstrated when viewed longitudinally (Fig. 1C). Viewing all seven LSCs together (Fig. 1A) highlights the onion-like appearance and the manner in which lamellae from different intricately interlaced LSCs. The different claw segments converge toward the central cleft. Each LSC extends a long segment directly through the cleft toward the axonal protrusions, from which other segments extend perpendicularly, forming the lamellar layers wrapping around toward the opposing side of the axon (Fig. 1, D and G). To further investigate the structural properties of the LSC cleft passage, we measured its branches within the cleft using the “Measurement Tool” in Webknossos. In total, the seven LSCs make 24 passages longer than 1 μm in the cleft (Fig. 1E). Of these, 18 lead to direct contact with the axon, while the other 6 do not. This suggests that LSCs use the clefts as “shortcuts” to interact with the axon. We also found that most LSCs (six of the seven reconstructed) enter only on one side of the cleft, and this can be either on the same side as the nucleus or on the opposite side (Fig. 1F).

Quantification of the surface area of each LSC revealed a pattern of significantly decreasing surface area from proximal to distal regions [*F*(7,48) = 11.88, *P < 0.0001, *n* = 7, one-way analysis of variance (ANOVA)], while the axon surface area conversely increased (fig. S4B). Together, we conclude that LSCs form stereotypical funnel-shaped, intertwined structures, which not only extend in a claw-like fashion but use the cleft as the main passage toward the axon.

### Contacts between LSCs and the afferent axon

**Fig. 2.**
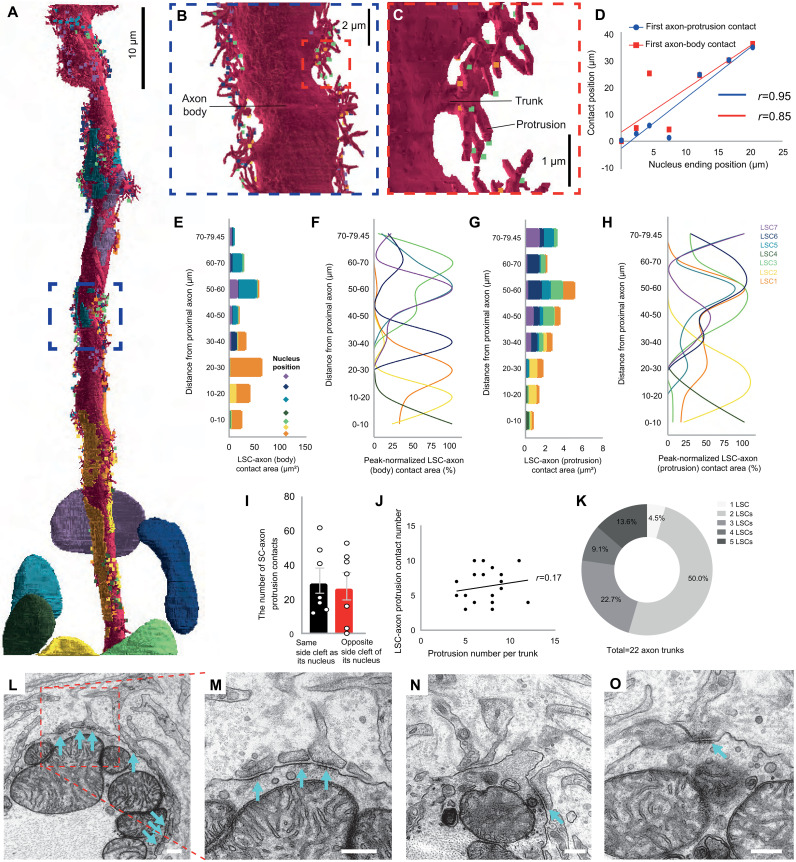
LSCs form contact points with the axonal body and protrusions. (**A**) 3D rendering of SBF-SEM reconstructions, showing the position of LSC nuclei, LSC-axon protrusion contacts (color squares), and LSC-axon body contacts (color patches on the axon body). (**B**) Higher magnification of the axon. (**C**) Higher magnification of the axon with an example trunk that sprouts into multiple protrusions. Green and orange squares show two LSCs making contact points on a group of protrusions. (**D**) Relationship between nucleus location and first contact point between LSC and axon. With Pearson’s correlation coefficients (*r*) both showing significant differences (*P* < 0.05). (**E**) Accumulating distribution (stacked bar chart) of LSC-axon contact areas on the axonal body along the length of the axon. (**F**) Peak normalized distribution of LSC-axon contact areas on the axonal body along the length of the axon. (**G**) Accumulating distribution (stacked bar chart) of LSC-axon contact areas on the axonal protrusions along the length of the axon. (**H**) Peak normalized distribution of LSC-axon contact areas on the axonal protrusions along the length of the axon. (**I**) Number of LSC-axon protrusion contacts, separated by LSC nucleus location. (**J**) The number of contact points on a trunk is independent of the number of protrusions that trunk has. (**K**) Number of LSCs making contact points on a single trunk. (**L**) Electron densities (cyan arrows) between the axonal membrane and LSCs tend to form clusters. Densities may be desmosomes or adherens junctions. (**M**) Higher magnification of (L). (**N**) Electron densities were typically found on or near axon protrusions and the cleft region [like in (L) and (M)]. (**O**) Example desmosome (blue arrow) between an LSC and the axonal membrane. Intermediate filaments can be seen proceeding inward from the axon membrane electron density. Scale bar for (L) and (M) is 200 nm.

The close proximity and tight claw-like wrapping of inner core LSCs suggests that they may have a direct role in potentiating mechanotransduction in PCs. Handler *et al.* (*4*) demonstrated that LSCs are structurally connected to the afferent terminal through a large number of protrusions distributed along its length and the main body. These protrusions are believed to serve as critical contact points and anchoring structures between the LSCs and the axon, forming a complex interface that is likely essential for mechanotransduction. This structural arrangement is proposed to facilitate the stretching of the axon and the subsequent activation of Piezo2 channels, enabling the effective transmission of mechanical forces from the surrounding lamellar layers to the axon (*4*). Therefore, revealing the direct contact points between LSCs and the axon, as well as providing detailed insights into the spatial and quantitative aspects of these interactions, is critical for advancing our understanding of mechanotransduction in the PC. Figure 2A depicts all the contact points (defined as a distance of 30 nm or less between axon and LSC; see Methods) made on both the afferent axon body and axonal protrusions (Fig. 2B). Along the axon, protrusions do not individually emerge directly from the axon; instead, they are grouped together and sprout out of common trunks (Fig. 2C). The distribution of LSC contact points has their highest density near the middle of our imaged volume and decreases sharply near the ultraterminal (fig. S4C). A previous study has shown that the protrusion density in the ultraterminal region is higher compared to the terminal region (*4*). Here, we demonstrate the possibility that the protrusion density changes along the axon. We observed that the position of the LSC nucleus determines its first contact with the axon (Fig. 2D), suggesting that there is a sequential order for the distribution of the individual LSCs and their contact with the axon. LSC-axon contacts are highly clustered, with each LSC having a preferred region where the axon body is contacted directly (Fig. 2, E and F) or on the trunks and protrusions (Fig. 2, G and H). Overall, LSCs have greater contact surface area with the axon body, but more numerous contact points on the axon protrusions (fig. S4D and table S1). Although the amount of LSC-axon protrusion contacts is regardless of the nucleus position relative to the cleft (Fig. 2J), we found a consistent number of LSC-axon protrusion contacts across trunks (on average six to seven contacts) on a local scale (Fig. 2K). Yet surprisingly, over half of these LSC-axon protrusion contacts were formed by only one or two individual LSCs (Fig. 2L). This means that contact points are not only regionally clustered but extremely selective, and comprise very few LSCs.

Next, we asked if these contact points contain any specific structural or functional elements. For this purpose we used a cryo-embedding approach (see Methods), which better preserves the membranes allowing different complexes to be more easily identified in TEM. We found electron-dense structures within LSC-axon contact zones (<30 nm apart) (Fig. 2, M to P), which tended to form multiple contact clusters at the cleft/axonal protrusion regions (Fig. 2, M to O). These junctions appear similar to those proposed to be adherens junctions (Fig. 2, M to O) (*4*) or desmosomes (Fig. 2P) (*24*). To estimate the number of junctions between LSCs and the axon, we took a stereological approach and counted the number of the above identified structures across eight groups of serial TEM images covering a total length of approximately 4.85 μm along the axon of one PC (PC1) and 4.65 μm of a second PC (PC2), and an axonal surface area of 83.5 and 62.5 μm^2^, respectively. We counted 146 and 134 junctions in PC1 and PC2, respectively, totaling 34.6 and 33.2 junctions/μm of axon length, and 2.15 and 2.85 junctions/μm^2^ axonal surface area. We extrapolated the number of junctions to a measured mean axon length of 226.54 μm, allowing an estimate of ~7523 to 7847 structural junctions along the length of a PC axon terminal. Among ~60 LSCs, this suggests that each LSC makes ~125 to 131 junctions with the axon. These structural junctions appear to keep the LSCs in close “contact” with the axon, but we could not find any evidence for gap junctions or synaptic and vesicular structures between LSCs and the axon. Together, these data suggest that LSCs are structurally tightly linked to the axon, with no obvious elements for classical functional coupling.

### Contacts between individual LSCs

**Fig. 3.**
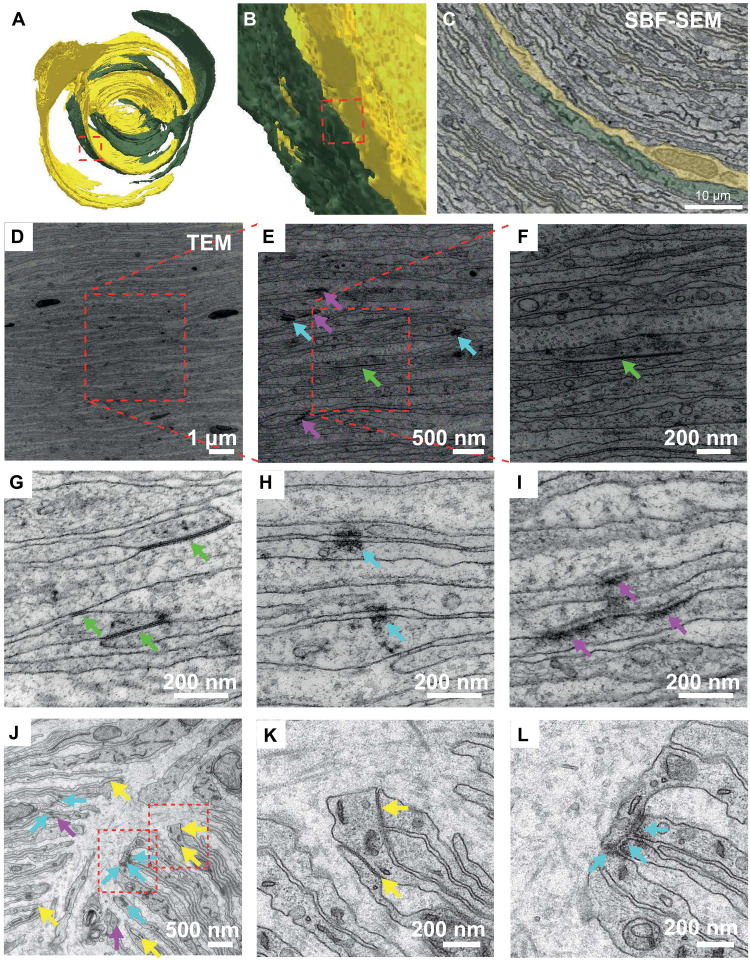
LSCs make gap junctions with other LSCs. (**A**) 3D renderings of two LSCs (LSC2 and LSC4). The red box denotes the location of an LSC-LSC contact (distance of less than 30 nm between two membranes). (**B**) Close-up of the site of the LSC-LSC contact in (A). (**C**) SBF-SEM image showing the contact between LSC2 and LSC4. (**D** to **F**) Cryo-temperature staining TEM of increasing magnification indicating the appearance of electron-dense structures including gap junctions (green arrows), desmosomes (cyan arrows), and hemidesmosomes (pink arrows). (**G**) Gap junctions (green arrows) are defined by symmetrical close appositions of thin electron-dense structures with no space between the membranes. (**H**) Desmosomes (cyan arrows) are marked by short symmetrical electron-dense element with a visible gap. (**I**) Hemidesmosomes (pink arrows) are electron-dense structures only found asymmetrical on a single membrane oriented toward the extracellular space. (**J**) Examples of groups of three to four LSCs, which tend to sit closely together and form concentrations of junctions at the inner core cleft region. We identified desmosomal-like structures (blue arrows), hemidesmosomes (pink arrows), and tightly opposed membranes, which are likely gap junctions (yellow arrows), although we could not confirm their precise identity in these samples. (**K**) Higher-magnification example of multiple likely gap junctions (yellow arrows) forming in clusters between LSCs at the cleft region. (**L**) Higher-magnification example of multiple desmosomes forming in clusters between LSCs at the cleft region.

Given the tightly packed nature of LSCs within the inner core, we next investigated the potential connections between individual LSCs. Previous ultrastructural studies reported the presence of tight junctions (*24*) and gap junctions between LSCs (*25*, *26*), suggesting that LSCs may reciprocally influence the electrical properties of adjacent LSCs. However, neither the density nor the distribution of either element is known. As these were not visible in the images taken with SBF-SEM (due to the embedding approach as well as the lower imaging resolution), we quantify instances of close apposition between LSC membranes as a means of quantifying potential contact areas (Fig. 3, A to C). By using the above described cryo-embedding approach, we were able to clearly identify a high density of desmosomes as well as gap junctions between LSCs (Fig. 3, D to I), which tend to be densely clustered between multiple LSCs joining at focal points at the cleft region (Fig. 3, J to L). To estimate the number of gap junctions and desmosomes within the inner core of the PC, we counted the number of these identified structures across serial, high-resolution images (2 nm/pixel) that covered an area of 680 μm^2^. We found the density of gap junctions to be 29.4/1000 μm^3^ and 41.2/1000 μm^3^ for desmosomes. We extrapolated these densities to the entire inner core volume of PC1, 140,900.72 μm^3^, and therefore estimate a total of ~4142 gap junctions and ~5805 desmosomes connecting the approximately 60 LSCs. This would mean that each LSC has on average ~69 gap junction and ~97 desmosome contacts to other LSCs. Together, these results suggest that the densely intertwined LSCs form not only a dense mechanical network but also a functionally tightly coupled network.

### Contact surface area between LSCs

The density of gap junctions between LSCs, but lack of gap junctions between LSCs and the axon, led us to ask the question if gap junction and desmosome densities are related to the total available contact surface area between LSC-LSC and LSC-axon contacts. We found that the number but not the area of LSC-axon protrusion contacts (fig. S5, A and B) and the LSC-LSC contact area (fig. S5C) appear to depend on the size of the LSC surface area (fig. S5D). Furthermore, we observed that most LSC-LSC contacts have similar contact surface area over the proximal to distal axis (fig. S6, A and B), but some individual LSCs have a greater overall LSC-LSC surface area (such as LSC6). Together, LSC-LSC contacts appear to maximize the total surface area between them, which may increase the probability for gap junction connections and thus rapid and high-fidelity LSC-LSC communication. In contrast, LSC-axon connections appear to maximize the number of contacts onto the axonal protrusions, and these contacts prioritize mechanical rather than functional coupling evidenced by high densities of desmosomes and an absence of gap junctions.

### LSCs and the vibration coding

**Fig. 4.**
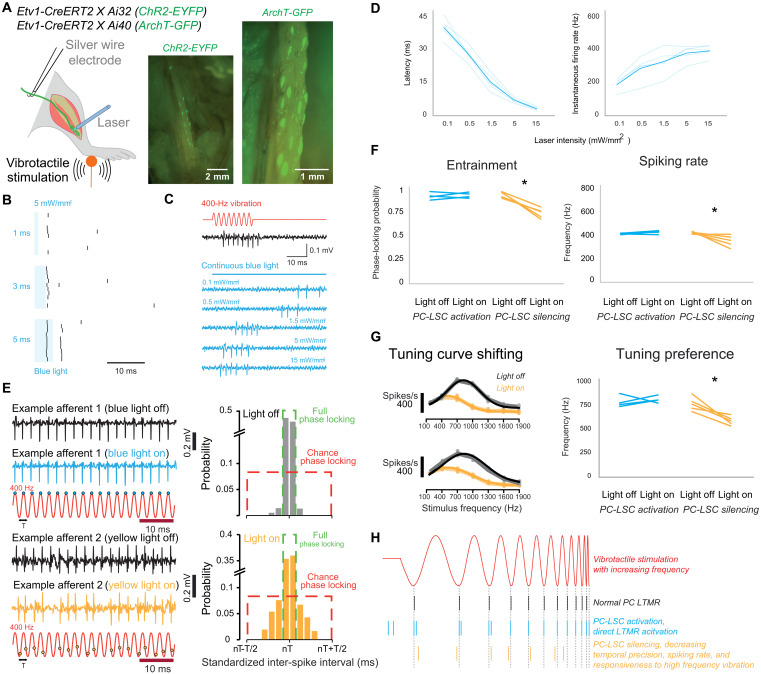
Optogenetic excitation and inhibition of LSCs in PC. (**A**) Schematic of exposed optogenetic activation of PCs during afferent recording (left). PCs on mouse fibula (each bright green spot is one PC), identified by EYFP or GFP (green fluorescent protein) expression in the inner core structures (green), were exposed after pulling skin and muscle aside (right). (**B**) With blue laser power at 5 mW/mm^2^, the latency of light-driven spiking of the afferent is very consistent regardless of stimulus pulse duration. (**C**) Example of spiking from PCs stimulated by blue light versus a mechanical stimulus. (**D**) Intensity-dependent activation of five afferents. Left, first spike latencies for PC afferents from optogenetic activation of LSCs; right, instantaneous firing rate during optogenetic activation of LSCs. (**E**) Left, example recording of two afferents (top, ChR2^+^; bottom, ArchT^+^) in response to 400-Hz hindlimb vibration, with light off (black) or on (blue/yellow). The timing of action potentials (blue/yellow dots) relative to periodic cycles of the 400-Hz vibration (red) shows the degree of entrainment. Right, with yellow light stimulation, the distribution of standardized inter-spike intervals of an example afferent at 400-Hz vibration shows decreasing degree of phase-locking probability. (**F**) Left, phase-locking probability of afferents affected by optogenetic activation or inactivation of PC-LSCs (paired-sample *t* test, *P < 0.0001). Right, spiking rate of afferents affected by optogenetic activation or inactivation of PC-LSCs (paired-sample *t* test, *P < 0.0001) in Etv1-ChR2 (*n* = 4) and Etv1-ArchT mice (*n* = 5). (**G**) Left, tuning curve shifting of two example afferents after inactivation of PC-LSCs by yellow light. Right, frequency tuning of afferents affected by optogenetic activation or inactivation of PC-LSCs (paired-sample *t* test, *P < 0.001). (**H**) Schematic model showing how PC-LSCs contribute to the coding of vibration. Additional spikes from PC-LSC activation (blue) illustrate enhanced excitability of the PC-LTMR.

To probe the functional impact of this connected network of LSCs on the axonal activity in PCs, we generated mice expressing channelrhodopsin (ChR2) or Archaerhodopsin-3 (ArchT) in Etv1-positive LSCs to excite or inhibit these cells with light (Fig. 4, A and B). We first used blue light to selectively activate LSCs in the PCs on the fibula while making single-unit recordings from PC-innervating Aβ fibers of the sciatic nerve, known as PC low threshold mechanoreceptors (PC-LTMRs). In Etv1-ChR2 mice, blue light reliably evoked ultra-short latency spikes in nearly 90% of the PC-LTMR (14 of 16) and other afferent types, including high-threshold mechanoreceptors, rapidly adapting receptors, and hair follicles, if the light was directed to their receptive fields (fig. S7). Light stimulation of LSCs evoked intensity-dependent spiking of the PC-LTMR (Fig. 4, C and D). Consistent with previous studies (*5*, *14*), the activation of nociceptive Schwann cells or sensory Schwann cells is also sufficient to trigger nerve electrical firing. PC-LTMRs recorded from Etv1-ChR2 mice respond faster to light stimulation than to vibration with a piezo actuator (fig. S7C). This broad range of first spike latencies to blue light stimulation (maximum up to 40 ms) was never observed in case of mechanical stimulation. The spike latencies (as a function of light intensity) in Schwann cells appear to be very different from those in cortical neurons, which are normally much shorter [5 ms; (*27*)], suggesting a complex mechanism in between the activation of the LSCs and the subsequent activation of the axonal afferent. On the other hand, the extremely short minimal latencies for the intense light activation suggest a tight physiological coupling between Etv1^+^ LSCs and the PC receptor ending.

To evaluate the contribution of LSCs to mechanosensitivity in PC-LTMRs, we compared the activation by vibrotactile mechanical stimuli in the same neuron with or without light-evoked activity (Fig. 4, E to G). With bidirectional optogenetic manipulation of LSCs in PCs, we discovered that the temporal precision spiking, spiking rate, and the tuning preference were all decreased in the case of LSC inhibition. With the decreased activity in LSCs, PC-LTMRs not only dropped their precise temporal code for vibration but also shifted their peak response to lower frequencies compared to the normal PC-LTMR (*28*), suggesting that the membrane potential of LSCs in the PC is critical for high-frequency vibration sensing (Fig. 4, E to G). Together, our results show that LSCs play a critical role in neural coding of PCs (Fig. 4H).

### LSCs and ultrasensitivity of perceptual thresholds for vibration

**Fig. 5.**
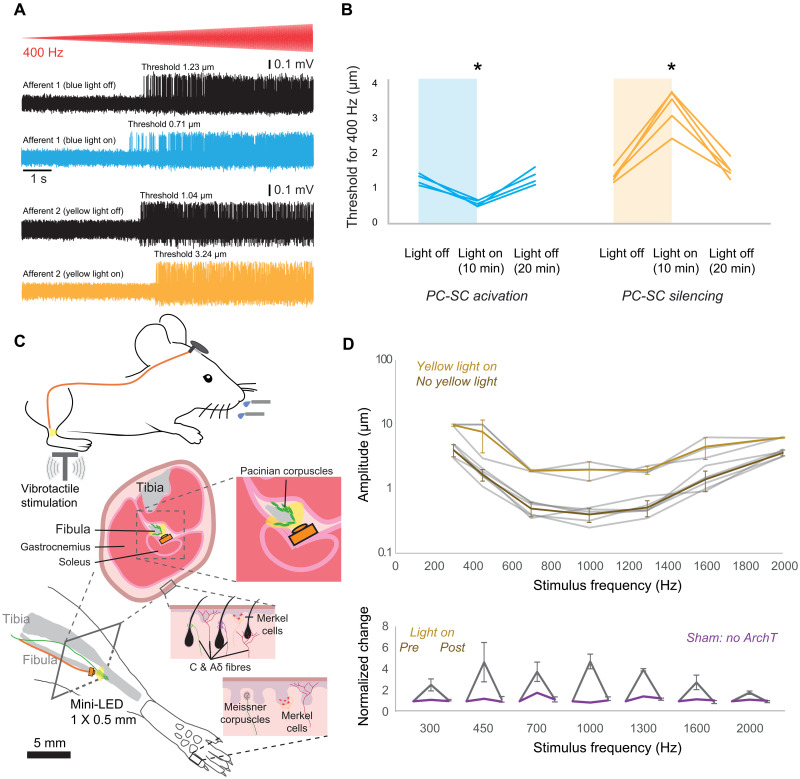
Schwann cells determine the perceptual threshold for vibration. (**A**) Mechanoreceptor spiking rates in response to 400-Hz vibration stimulus before and after optogenetic activation or inhibition of Schwann cells. (**B**) Mechanical threshold for first spike for afferents recorded in Etv1-ChR2 (*n* = 4) and Etv1-ArchT mice (*n* = 5). A decrease in the force necessary to evoke the first action potential was observed in afferents recorded from Etv1-ChR2 mice, but the effect was revered in Etv1-ArchT mice. One-way ANOVA, *P < 0.05, Bonferroni’s multiple comparisons test (two sided). (**C**) Schematic showing behavioral setup with hindlimbs placed on a vibrating platform. During and after the light exposure with implanted mini-LEDs on fibula, their sensitivity to the vibrotactile stimulation was tested again. The LEDs were attached to the bone and placed under the soleus muscle as shown in the cross-section illustration. (**D**) Top, v-shaped perceptual sensitivity curves before and during the optogenetic inhibition. Amplitude thresholds as a function of vibration frequency for mice (shaded lines: individual mice, symbols: mean). Bottom, the behavioral effect of PC-LSC inhibition at individual frequencies. One-way ANOVA, *P* < 0.05, Bonferroni’s multiple comparisons test (two sided).

We next used light-induced activation and silencing to ask if Etv1^+^ LSCs within PCs contribute to the ultrasensitivity of vibrotactile stimuli. In Etv1-ChR2 and Etv1-ArchT mice, we evaluated the sensitivity using a 400-Hz sinusoidal stimulus with a linearly increasing amplitude (Fig. 5A). We measured the amplitude of vibration for the first spike before and after 10 min of continuous blue and yellow light was focused on the corpuscles. This method was used in the previous studies to demonstrate the physiological impact of Schwann cells on the sensitivities of various mechanoreceptors (*5*, *14*). In Etv1-ArchT mice, all of the PC-LTMRs showed a significant elevation in mean mechanical threshold during light stimulation compared to baseline (Fig. 5B). Our data thus support the hypothesis that Etv1^+^ LSC activation within PCs lowers the threshold and increases vibration sensitivity of the corpuscles.

Next, we examined the role of Etv1^+^ LSCs within the PC in regulating the perceptual thresholds of mice in a vibrotactile detection task. We adapted a tactile perception task for water-restricted, head-restrained mice (*29*), in which Etv1-ArchT mice were trained to report a high-frequency vibration stimulus delivered to the hindpaw (Fig. 5C; see Methods for details). Following 12 to 25 days of behavioral training, mice could correctly report detection of the stimulus by licking a water spout within a 2-s response time window (fig. S8). After training, we implanted mini-LEDs (light-emitting diodes) into the fibula region of both hindlimbs, controlled remotely through a custom-made wireless optogenetic device [BlueBerry (*30*); see Methods for details], to inhibit Etv1-ArchT^+^ LSCs in PCs during behavioral tasks. After a few days of recovery, we retested the mice in the perceptual task during light exposure. The detection tasks yielded comprehensive sensitivity curves, and mice with LSCs inhibited by exposure to yellow light showed a reduction by a factor of 5 in their ability to correctly detect the stimulus across all frequencies (Fig. 5D). This effect was reversible, as post-exposure tests showed that the mice had recovered their perceptual performance back to control levels (Fig. 5D). To test whether the effects of yellow light itself, in the absence of ArchT, could activate LSCs, we trained an additional mouse that lacked ArchT expression (Fig. 5D, bottom). Consistently, the mouse showed no changes to its perceptual threshold following an identical procedure of yellow light exposure of the hindlimbs. Finally, although we found expression of Etv1 associated with other receptor types in the skin (fig. S9), our histological evidence suggests that Etv1 is expressed selectively in inner core LSCs. We could not completely rule out Etv1 expression in the axon, as 1 of 11 PCs had a small tdTomato fluorescence intensity rise in the axon; however, it was extremely low in comparison to the inner core region (fig. S10). Because of the positioning of the mini-LED over the PCs and blocked from the skin by overlying muscles (Fig. 5C), this suggests that we were able to selectively inhibit the PC LSCs, without influencing other cell types. Together, these data demonstrate that LSCs in PCs are essential to achieve the lowest threshold levels for high-frequency vibrotactile stimuli.

## DISCUSSION

PCs are essential for the detection of various types of externally and internally generated stimuli (*3*, *31*, *32*). We reveal their intricate, convoluted, multilayered, and connected morphology. Reconstructions of multiple cells show how their layers intertwine, and wrap around the axon in a hair-claw–like manner, consistent with a previous study (*4*) and the inner cores of avian PCs in the accompanying study (*33*). In those cases, the afferent contains dense protrusions, which extend through the cleft structure. A comprehensive understanding of the ultrastructural features of LSCs not only is crucial for refining our understanding of the physical properties of the PC but also will guide future experiments to study the developmental stages of PCs. Currently, very little is known about how PCs develop and how their function evolved. We also explored the relationship between the positions of the LSC nuclei and the spatial distribution of the LSC-axon contacts (Fig. 2, E to H) and, surprisingly, revealed a strong spatial correlation between the LSCs’ nucleus position and their first contact to the axon (Fig. 2D), which suggests a “first-come first-served” characteristic, potentially related to developmental mechanisms of the inner core (Fig. 2D). It is possible that the LSCs closer to the ultraterminal developed later than the LSCs closer to the preterminal (Fig. 1A), and the later-developed LSCs can only make contact with the axon in the distal region. This is consistent with the observation that the location of each LSC passage through the cleft also follows each LSC’s nucleus position (Fig. 1F).

The axon membrane is believed to be stretched by LSCs during mechanical stimulation, leading to the opening of axonal Piezo2 channels and subsequent neuron excitation (*4*). However, it remains unclear whether the mechanism by which the axon membrane is stretched differs between the axon body and the protrusion, and how the lamellar structures move to stretch the axon membrane. Here, we observed that the contact area between the LSC and the axon body is larger than that between the LSC and the axon protrusion (fig. S4D). The contacts between the LSC and the axon protrusions typically have multiple small contact points, and there is a trend toward a positive correlation between the surface area size and the number of axon protrusion contacts (fig. S5A). Building on Handler *et al.* (*4*), we estimate that each LSC makes ~125 to 131 structural junctions (either adherens or desmosomes) and these were particularly focused at the protrusion zones, where the LSCs make the small, targeted contact points. In contrast, the LSC does not merely contact the axon body; rather, the lamellar structure resembles a sheet that adheres to the axon body (Fig. 2A). Here, the adherens junctions and desmosomes likely act as anchoring points, either facilitating the transmission of mechanical forces from the outer capsule to the central axon or keeping the membranes in close apposition for some other form of communication, as suggested above. Further investigation is needed to determine how the activation of axonal Piezo2 channels in different locations of the axon contributes to neuron excitation under these circumstances.

Other than making contact with the axon, a major feature of LSCs is the densely packed thin lamellae (53 layers on average). Our observation that LSC-LSC contacts can be predicted by the surface area of two LSCs (fig. S5) suggests that direct communication (gap junctions) between LSCs can be linearly increased by enlarging the surface area, resulting in these highly convoluted structures. Our results allow an estimation of ~69 gap junctions and ~97 desmosomes per LSC (totaling ~4142 gap junctions and ~5805 desmosomes in the whole inner core), suggesting that LSCs appear to maximize their contact areas with each other for rapid high-fidelity functional connections. In contrast, the lack of gap junctions between LSCs and the axon suggests prioritization of mechanical interactions with the axon. This is well supported by the accompanying study (*33*), which demonstrates that injection of the gap junction permeable dye, lucifer yellow, into inner core LSCs permeates all of the inner core lamellar cells but does not permeate into the axon. Therefore, densely packed lamellae might not only facilitate the transmission of physical vibration (*3*, *34*) but also, at the same time, enhance the information exchange within these complex networks of LSCs for rapid, high-fidelity, and synchronous communication.

Previous studies have shown that Schwann cells actively modulate nerve activity and regulate mechanical nociception (*5*, *14*), suggesting that terminal Schwann cells are not only supportive but also crucial for directly modulating mechanosensation. Moreover, voltage-sensitive Na^+^ channels (*35*) and acid-sensing ion channel 2 (ASIC2) (*36*) have been identified in the inner lamellae of PCs, where LSCs are located, suggesting their role in action potential generation. In addition to Piezo2, other channels, such as ASIC2 (*36*) and the β-ENa + C and γ-ENa + C subunits (*37*), have also been identified in the axon, indicating that additional mechanisms may contribute to mechanotransduction.

Our electrophysiological and optogenetic experiments suggest that LSCs within PCs may influence both neural coding of vibrotactile stimuli and the perceptual threshold for vibration. Specifically, optogenetic inhibition of a subset of LSCs disrupted the temporal precision, spiking rate, and tuning preference of Pacinian afferents while also elevating the threshold for vibration detection—an observation consistent with previous work indicating that Schwann cells in Meissner’s corpuscles are critical for low-threshold vibrotactile perception (*5*). However, we acknowledge that these insights derive from optogenetic manipulation and may not fully reflect normal physiological conditions in the absence of artificial stimulation. The lamellated architecture of Pacinian LSCs, presented in this and the accompanying study (*33*), raises an important question: What are the mechanisms by which these complex LSC structures influence the physiological responses of PCs? Previous research has suggested that LSCs may release neurotransmitters to regulate axonal activity and mediate rapid adaptation (*13*). However, the anatomical and functional evidence of LSC-LSC gap junctions, described above, suggests that LSCs act in concert to influence the axon terminal. In addition to the previously proposed mechanical interactions (*4*), this raises the possibility of other mechanisms such as ephaptic coupling generating local field potentials across the LSCs and increasing LSC and/or axon excitability. We propose future in vivo experiments to visualize membrane activity and physical movements during vibrotactile and optogenetic stimulation. Such experiments could greatly advance our understanding of how LSCs participate in the complex process of mechanotransduction and the exquisite vibration frequency tuning of PCs.

Etv1 is a transcription factor that has been found in various cell types, including the heart (*38*), taste cells (*39*), proprioceptive afferents, a small subset of motor neuron pools in the lumbar spinal cord (*40*), and glial cells (*41*). In our experiments, we locally shed light near the fibula region of both hindlimbs, where only the PCs reside, ensuring that no other types of mechanoreceptors were affected. The perceptual change upon optogenetic inhibition is precisely aligned with the known vibration sensitivity of PCs (Fig. 5) and below the sensitivity range of other afferent types. In 1 of 11 PCs, we observed tdTomato expression in an afferent of a PC; however, the fluorescence intensity level was only measurable when the surrounding LSCs’ fluorescence was saturated, suggesting extremely low comparative expression levels (fig. S10). Therefore, the consistent physiological results from our optogenetic experiments are better explained by the activation/inhibition of LSCs (Figs. 4 and 5).

Finally, we demonstrated reduction in the neuronal vibration detection thresholds upon optogenetic activation of LSCs, which is partially consistent with the accompanying study that showed that electrical activation of LSCs decreases the threshold of mechanical activation of the avian Pacinian afferent (*33*). Additionally, we demonstrate the elevation of vibrotactile perceptual thresholds by a factor of 5 upon optogenetic inhibition of hindlimb LSCs (Fig. 5), suggesting that LSCs in PCs are essential for detection of vibrotactile stimuli at the lowest perceivable vibration amplitudes. Overall, our results establish LSCs as a critical player in the exquisite sensitivity to high-frequency vibrotactile stimuli, facilitated by PCs (*33*). In the future, it will be interesting to investigate if higher vibrotactile sensitivity thresholds could affect gait, locomotion, dextrous motor control, and bodily self-awareness (*32*).

## METHODS

### Animals

All electron microscopy experiments were carried out in double-transgenic mice. Homozygote Ai14 males carrying a floxed tdTomato fusion gene inserted in the Gt(ROSA)26Sor locus in a C57BL/6 strain (*42*) (Jackson Laboratory, stock no. 007914) were mated with heterozygote ER81/Etv1-CreER females expressing the CreERT2 fusion protein from the ER81/Etv1 promoter elements (*43*) (Jackson Laboratory, stock no. 013048). The Cre-mediated recombination resulted in the expression of the floxed tdTomato sequence in the ER81/Etv1-expressing cells of the offspring. Among other cells, ER81/Etv1 is expressed in the inner core region of PCs (*44*). To induce CreER-based recombination, double-transgenic adult offspring were administered five consecutive daily doses of 100 μl of tamoxifen (T5648, Sigma-Aldrich) solution (20 mg/ml) dissolved in corn oil (C8267, Sigma-Aldrich) by peritoneal injection. Electrophysiology experiments on nerve afferents were conducted with C57BL/6 (Charles River Laboratory) mice. Wild-type mice were purchased from Charles River, and transgenic mice were obtained from The Jackson Laboratory and bred in the animal facility of the University of Geneva. Experiments involving optogenetic manipulation of Schwann cells were conducted in double-transgenic mice generated by mating homozygous Ai32 mice carrying a floxed *ChR2*-EYFP (enhanced yellow fluorescent protein) fusion gene inserted in the *Gt(ROSA)26Sor* locus in a C57BL/6 strain (Jackson Laboratory; stock no. 012569) or homozygous Ai40 mice carrying a floxed *ArchT*-EYFP fusion gene inserted in the *Gt(ROSA)26Sor* locus in a C57BL/6 strain (Jackson Laboratory; stock no. 021188) with heterozygote ER81/Etv1-CreER mice.

The mice were housed in an animal facility in groups of maximum five animals per cage and maintained on a 12-hour:12-hour light/dark cycle. All experiments were performed during the light phase of the cycle. The animals did not undergo any previous surgery, drug administration, or experiment. All procedures were approved by the Institutional Animal Care and Use Committee of the University of Geneva and Geneva veterinary offices.

### Surgery

Fifteen- to 30-week-old mice were surgically prepared for terminal electrophysiology. Surgeries were conducted under isoflurane anesthesia (1.5 to 2%), and additional analgesic [buprenorphine (0.1 mg/kg), intramuscular (im)], local anesthetic [75 μl of 1% lidocaine subcutaneous (sc) under the location for incision], and anti-inflammatory drugs [dexamethasone (2.5 mg/kg, im) and carprofen (5 mg/kg, sc)] were administered. Mice were fixed on a bite bar with a snout clamp and rested on top of a heating pad. All electrophysiology experiments were surgically prepared under terminal anesthesia by inhalation of isoflurane (~2%), and the body temperature was maintained near 37°C. For analgesia, buprenorphine (0.1 mg/kg, sc) was provided 15 to 20 min before the procedure, except for behavioral experiments, where chronic preparation was used. An additional dose of buprenorphine was given if the procedure exceeded 4 hours. The whole procedure was limited to 8 hours. For chronic optogenetic experiments, a custom-made titanium head bar and a small headstage for wireless multichannel optogenetic system were fixed on the skull with a cyanoacrylate adhesive (ergo 5011, IBZ Industrie) and dental cement to allow subsequent head fixation. After exposing the PCs with a small incision, mini-LEDs were sutured to the tissue right on top of the fibula bilaterally and were connected to the headstage with insulated bronze cables threaded underneath the skins (*45*). The incision then was closed by suturing. After the initial surgical phase, the animal was maintained under light isoflurane anesthesia (0.25 to 0.75%) and allowed at least 2 days of recovery before recording.

### Electrophysiology

For electrophysiology experiments, mice were anesthetized by isoflurane inhalation (2%) and body temperature was maintained near 37°C with a heating mat. For analgesia, buprenorphine (0.1 mg/kg, sc) was provided 15 to 20 min before the procedure. To assess the sensory afferents innervating PCs, a skin opening was performed along the hamstring muscle. Then, the sciatic nerve was carefully isolated using ophthalmic scissors and tweezers. The pia mater spinalis and dura mater around the nerve were removed, and finally, the nerve bundle was separated into single fibers (15 to 20 μm in diameter) and placed on two Ag/AgCl wire electrodes in the recording pool filled with mineral oil. A third ground Ag/AgCl electrode was placed in the muscle next to the recording chamber.

To characterize the response properties of sensory afferents at the peripheral level, we recorded stimulus-evoked activity of afferents in vivo using hook electrodes in the tibial nerve. Vibratory stimuli were applied to different locations of the hindlimb or forelimb using a calibrated piezo stimulator (Physik Instrumente P-841, E509 controller, E504 amplifier) so that the neural responses to all combinations of locations, frequencies, and amplitudes could be characterized. To determine the mechanical sensitivity threshold and frequency tuning of each afferent, a series of sinusoidal mechanical stimuli (duration of 20 s for frequencies above 100 Hz; linearly increasing amplitude) were applied to the hand-mapped receptive field of individual afferents with the piezo actuator. To study purely vibration induced responses, the probe was first placed on the skin for a period of time of a few seconds to minutes and the stimulation for threshold mapping was slowly increased. Alternatively, thresholds for each frequency were defined as the amplitude required to elicit 20% of a unit’s maximum firing during the sustained part of the stimulus (200-ms window beginning 50 ms after onset), as was used in Huey *et al.* (*46*).

The signal was amplified and filtered (>10 Hz and <10 kHz) and acquired at 30 kHz (PXIe-1073, National Instruments) using WaveSurfer (https://wavesurfer.janelia.org/) Matlab (MathWorks) routines. Trial start, stimulus onset triggers, and details of stimulus were saved in parallel on separate channels and used for post hoc alignment of recorded spikes. After the recording, animals were euthanized by overdose with isoflurane (5%) followed by cervical dislocation and bleeding.

### Vibrotactile stimuli

The vibrotactile stimuli were generated by a bimorph piezoelectric multilayer bender actuator with a piezoelectric stack actuator (P-841.3, Physik Instrumente) equipped with a strain gauge feedback sensor for high-frequency stimulations (above 100 Hz). A hand-cut blunt plastic cone was mounted on the actuator. The actuator and sensor controllers (E-662, E-618.1, and E-509.S1, Physik Instrumente) were operated in an open loop with a contacting force below 50 mN on the mouse skin. The forces of our vibrotactile stimuli measured at the static states of 3 and 30 μm were less than 10 mN and 30 mN, respectively. Pure sinusoids (250- or 500-ms duration) of a wide range of frequencies (100 to 1900 Hz) calibrated to produce a desired displacement amplitude (0.01 to 20 μm) were sampled at 10 kHz (USB-6353, National Instruments) and fed to the actuator controller. The sensor measurements were continuously acquired, and recalibration of motor commands was regularly performed for the stimuli to remain highly consistent, by comparing the ground-truth data acquired optically with a laser Doppler vibrometer system (Polytec, OFV-5000). The spectrum of the acquired sensor measurements was analyzed to ensure the integrity of their frequency content (*47*, *48*).

### Optogenetic stimuli

Optogenetic stimulation of Schwann cells in PCs was generated by blue light illumination (473 nm, 50 mW; Obis LX FP 473, Coherent) out of a fiber [3.5-μm core diameter, 0.045 numerical aperture (NA)] operated in analog control mode or by yellow light illumination (568 nm, 500 mW; Sapphire LP/LPX, Coherent) fed into an optic fiber with achromatic FiberPorts (Thorlabs), gated with a shutter. The laser power or the shutter was controlled by analog signals from the WaveSurfer (https://wavesurfer.janelia.org/). For the blue laser, the stimulus was a continuous square pulses (2000-ms duration). The mean power of the laser for continuous stimulation used in our experiments was 0.3 mW for blue and 0.5 mW for yellow (measured at the tip of the fiber).

We used a custom-made wireless multichannel optogenetic system (BlueBerry) (*30*) to apply the stimulation through mini-LEDs (0402 SMD-QBLP595-IG) in the fibula region. The BlueBerry is made of a low-power Bluetooth module (RN4871-microchip) that communicates the stimulation protocols (frequency, duty cycle, LED channel) with a microprocessor (ATTINY 85) connected to both mini-LEDs. A mobile application (BLE Terminal) is used to transmit all the stimulation parameters to the Bluetooth module of the BlueBerry. For optical stimulation of PCs, the stimulus was 3-ms short square pulses flashing in 20 Hz with approximately 3 mW.

### SBF-SEM and TEM: Euthanasia, perfusion, and dissection

Adult mice (15 to 30 weeks) were anesthetized with sodium pentobarbital (60 mg/kg) by intraperitoneal injection. They were subsequently transcardially perfused with 15 ml of 0.1 M phosphate-buffered saline (PBS), followed by 60 ml of 2% paraformaldehyde (PFA; 15710-S, Electron Microscopy Sciences)–1% glutaraldehyde (G7526, Sigma-Aldrich) in 0.1 M PBS (pH 7.4). The perfused animals were placed in a sealed plastic bag for 1 to 2 hours before the dissection of PCs was carried out. Dissection of PCs (fig. S1) was carried out under a fluorescent stereomicroscope (Leica M165FC). First, the four limbs were isolated by cutting above the elbow/knee and the skin was cut away until the wrist/ankle. The paw was pinned onto a dissection tray with the palm/sole facing up, and then muscles and tendons were gently cut away to reveal the ulna or fibula and tibia. To identify the PCs, the expression pattern of tdTomato was revealed by illuminating the bone green LED light and using an ET TxRed filter set (Leica). The PCs were present in bundles (resembling a cluster of grapes) and could be dissected from the periosteum by gently lifting them off the bone with fine forceps (Dumont #5SF Forceps, FST) and pulling the bundle up where the afferents converge. Once dissected, the PCs were postfixed overnight at 4°C, by immersion in 2% PFA (15710-S, Electron Microscopy Sciences)–1% glutaraldehyde (G7526, Sigma-Aldrich) in 0.1 M PBS (pH 7.4).

### SBF-SEM: Sample processing and imaging

For SBF-SEM, the sections were postfixed, stained, and embedded according to a protocol similar to Hua *et al.* (*49*). This was done by the BioEM Facility of the Ecole Polytechnique Federale Lausanne (EPFL). In brief, the samples were first postfixed in 1.5% potassium ferrocyanide and 2% osmium in 0.13 M ice-cold cacodylate buffer. Then, they were stained sequentially by 1% thiocarbohydrazide (40 min at 40°C), 2% osmium tetroxide (90 min at room temperature), 1% uranyl acetate (overnight at 4°C), and finally lead aspartate solution (120 min at 50°C). This was followed by dehydration in increasing concentrations of ethanol (50%, 70%, 96% × 2, 100% × 2) and then embedding in Durcupan resin. The samples were left to harden at (65°C).

Sample quality was confirmed, and regions of interest were identified using TEM. The blocks were then trimmed and glued to an aluminum stub using conductive resin. The imaging was carried out in a scanning electron microscope (Zeiss Merlin, Zeiss NTS) containing an ultramicrotome (3View, Gatan), also at the BioEM Facility of the EPFL. Two PCs were imaged. For both, an acceleration voltage of 1.7 kV and a dwell time of 1 μs were used. An acceleration voltage of 1.7 kV was used with a pixel size of 6.5 nm and a dwell time of 1 μs. Two PCs were imaged. For the first (“PC1”), a pixel size of 40 nm was used, and the resin slice cut between each image had a thickness of 200 nm. The total size of the block was 138 μm × 145 μm × 273 μm. It contained a full PC. For the second PC (“PC2”), a pixel size of 7 nm was used, and the resin slice cut between each image had a thickness of 50 nm. The total size of the block was 29 μm × 31 μm × 80 μm.

### SBF-SEM: Image processing

Initially, image stitching and alignment was done in the TrakEM extension of FIJI imaging software. For PC1, two tiles were acquired; these were stitched using the “Montage multiple layers” feature of TrakEM. Then, the “Align layers” feature was used to align all layers, for both PC1 and PC2. Where necessary, small manual adjustments were made. These images were then exported into the software Amira (Thermo Fisher Scientific), which was used for segmentation. For PC1, the axon, inner core region, nuclei of inner core cells, intermediate layer region, 14 outer core layers, capsule region, and myelin sheath were reconstructed (fig. S2). For PC2, the axon (fig. S2), as well as an inner core cell (fig. S3), was reconstructed in a 25-μm subsegment. The segments were visualized in Amira using the “Generate Surface” tool.

### SBF-SEM: Machine learning–assisted image segmentation

To extract individual LSCs in this electron microscopy dataset, the stacks of electron microscopy images were first processed using automated segmentations via Webknossos automated segmentation services, followed by manual proofreading. Initially, a CNN was trained using our manual segmentations. Subsequently, the trained model was used to generate affinity predictions, indicating the likelihood of a voxel being connected to its neighboring segments. Volume segments were then grouped into agglomerates ranked based on their predicted affinity (*23*) with additional restraints to reduce the occurrence of merge errors (e.g., each segment contained only one nucleus). Furthermore, we conducted human proofreading to the fullest extent possible to rectify merge or split errors before proceeding with the data analysis of these annotations.

### TEM: Sample processing and imaging

After overnight postfixation, the samples were prepared for TEM as follows. Each sample was placed in a 10-ml glass test tube with a cork lid for the whole protocol. The samples were washed at room temperature in 0.1 M PB (pH 7.4). Then, they were postfixed in 2% osmium in the Millonig buffer for 1 hour at 4°C, followed by washing at room temperature with double distilled water (ddH_2_O). The samples were then stained overnight with 0.25% uranyl acetate at 4°C. The next morning, the samples were washed with ddH_2_O. They were then dehydrated in ethanol (15058, Reactolab SA), increasing concentration from 30%, 50%, 70%, 90% to finally 100%. This was followed by 100% propylene oxide (82320, Fluka Analytical), twice for 10 min. In the meantime, the epoxy resin was prepared. For 100 g, the following were mixed: 46.34 g of epoxy embedding material (45345-250ML-F, Sigma-Aldrich), 27.76 g of dodecenylsuccinic anhydride (DDSA; 45346-250ML-F, Sigma-Aldrich), 25.9 g of hardener methyl-5-norbornene-2,3-dicarboxylic anhydride (MNA; 45347-250ML-F, Sigma-Aldrich), and 1.5 g of accelerator (45348-250ML-F, Sigma-Aldrich). The samples were then left in a 50% propylene oxide and 50% epoxy resin mixture for 30 min and then left in 100% epoxy resin mixture overnight, both at room temperature. Finally, the samples were moved from the glass test tubes into a silicone mold with fresh 100% epoxy resin mixture. The samples were left to cure for 72 hours at 60°C.

The resulting resin blocks were trimmed, and ultrathin sections were cut at a thickness of ~60 nm using a diamond knife (DiATOME Ultra 45°) and ultramicrotome (Leica Ultracut UCT). The resin blocks were mounted in the ultramicrotome so that the PCs would be cut along the coronal axis. The cut slices were collected on slot grids that had been prepared at the Electron Microscopy Facility of the University of Lausanne. The sections were poststained with 5% uranyl acetate for 10 min and lead nitrate for 7 min.

Imaging was carried out on an FEI Morgagni transmission electron microscope. For an overview of the full PC, images were taken at a magnification of ×2800. To image the inner core, images were taken at a magnification of ×8900 to ×14,000. For higher resolution of details, images were taken at a magnification of ×28,000. When the region of interest was larger than the field of view, multiple tiles were taken to cover the whole area of interest.

### TEM: Image processing

Images were cropped and adjusted for optimal contrast using Fiji/ImageJ. If multiple tiles were taken, the Plan Brightness Adjustment plugin of Fiji was applied to all images, and then they were aligned and stitched together in the TrakEM extension of Fiji. The TEM images presented in this study were pseudo-colored by hand using Illustrator.

### Cryo-temperature staining and embedding for TEM

The perfused, chemically fixed samples were cryoprotected in a solution of 2% glycerol and 20% dimethyl sulfoxide (DMSO), in 0.01 M PBS, and then high-pressure frozen in the same solution (Leica MicroSystems ICE high-pressure freezer). They were then placed in plastic cryotubes inside a low-temperature embedding machine (AFS, Leica Microsystems) held at −90°C. These tubes contained acetone with 2% osmium tetroxide, 0.2% uranyl acetate, and 5% water. The temperature was then raised to −20°C over 3 days, and then the solution was exchanged for pure acetone while the temperature was raised again to 0°C. At this point, the acetone was exchanged for a 50/50 mixture of Epon resin and acetone, and the temperature was then brought up to 20°C. After several exchanges of pure resin, the samples were then flat embedded between glass slides and the resin was hardened for 24 hours at 65°C.

### Immunohistochemistry and anatomical characterization

Adult Etv1 × Ai14 (tdTomato) mice (15 to 30 weeks) were anesthetized with sodium pentobarbital (150 mg/kg; Eskonarkonad.us.vet., Streubli Pharma AG) by intraperitoneal injection. They were subsequently transcardially perfused with 15 ml of 0.1 M PBS, followed by 20 to 30 ml of 4% PFA (15710-S, Electron Microscopy Sciences) in 0.1 M PBS (pH 7.4). PCs were dissected out from the hindlimbs and forelimbs in bundles of 15 to 30 PCs and postfixed in 4% PFA for 20 min before rinsing 3 × 10 min in 0.1 M PBS. PC bundles were then embedded in 5% agarose (A5093, Sigma-Aldrich) in PBS and sliced in 50-μm sections using a vibratome (Leica VT1000S), before being left to dry at room temperature on microscopy slides (Superfrost Plus Adhesion Slides, J1800AMNZ, Epredia) for 2 hours, protected from light. For skin and muscle samples, hair was removed from the hindlimbs and skin and soleus muscles were dissected out. Skin and muscle were postfixed in 4% PFA overnight (4°C), followed by rinsing 3 × 20 min in 0.1 M PBS. Tissues were then left in 15% sucrose in 0.1 M PBS until they sank to the bottom of the tube, and then moved to 30% sucrose in 0.1 M PBS. After sinking, tissues were placed on microscope slides and excess solution was wicked away using a precision wipe (05511, Kimtech Science), before allowing to freeze in −80°C for 10 to 20 min. Tissues were mounted in the desired orientation in OCT (KMA-0100-00A, CellPath) and sliced at 30-μm thickness, using a cryostat, before mounting onto slides.

All tissue sections (PCs, skin, muscle) were allowed to dry at room temperature for 2 hours (protected from light) to adhere to slides, and then rinsed in 0.1 M PBS, 3 × 5 min. Sections were permeabilized 3 × 20 min in 0.3% Triton X-100 (T9284, Sigma-Aldrich) in 0.1 M PBS (PBST) with gentle shaking (50 rpm) and blocked for 2 hours with 0.1% PBST and 5% bovine serum albumin (BSA) before adding primary antibodies and incubating overnight at 4°C. Primary antibodies included protein gene product 9.5 (PGP9.5) (1:500, PA1-10011, Invitrogen) to label nerve fibers in all tissues, and skin sections were additionally incubated with guinea pig calcitonin gene–related peptide (CGRP) (1:500, 414004, Synaptic Systems), rabbit tyrosine hydroxylase (TH) (1:500, 213102, Synaptic Systems), or rat anti-cytokeratin 8 (TROMA-1) (1:200, MABT329, Sigma-Aldrich). Sections were washed 3 × 10 min in 0.1% PBST and incubated for 2 hours with secondary antibodies (Alexa Fluor 488 goat anti-rat A11006, Alexa Fluor 488 goat anti–guinea pig A11073, or Alexa Fluor 488 goat anti-rabbit A11008, and Alexa Fluor 647 goat anti-chicken A21449, Invitrogen) in 5% BSA (A3912, Sigma-Aldrich), in 0.1% PBST at room temperature. Sections were then washed 2 × 10 min with 0.1% PBST, incubated with 4′,6-diamidino-2-phenylindole (DAPI) for 5 min, and washed a further 3 × 10 min with 0.1 M PBS before exchanging with mounting medium (Fluoromount Aqueous Mounting Medium, F4680-25ML, Sigma-Aldrich) and placing coverslips.

Dorsal root ganglion (DRG) slices were dried at 37°C for 2 hours (protected from light) to adhere to slides, and then rinsed in 0.1 M PBS, 3 × 10 min. Sections were permeabilized 3 × 10 min in 0.1% PBST and blocked for 2 hours with 10% donkey serum (Abbkine, #BMS0140) before adding primary antibodies and incubating overnight at 4°C. Samples were incubated in primary antibodies overnight at 4°C to label DRG nociceptors CGRP (rabbit anti-CGRP, 1:300, Cell Signaling Technology, #14959) and TH (rabbit anti-TH, 1:300, Proteintech, 25859-1-AP), parvalbumin neurons (mouse anti-parvalbumin, 1:300, MAB1572, Sigma-Aldrich or 195011, Synaptic Systems), and sensory nerve fibers (mouse anti-NF200, 1:400, MAB5266, Sigma-Aldrich). After washing (3 × 10 min in 0.1% PBST), sections were incubated for 2 hours at room temperature with secondary antibodies (Alexa Fluor 488 goat anti-mouse A32723 or Alexa Fluor 488 donkey anti-rabbit A21206) (1:500) in 10% donkey serum. DRG slices labeled with IB4, 4 μg/ml IB4-FITC (fluorescein isothiocyanate-conjugated isolectin B4) (Invitrogen, catalog no. I21411) were put in dilution buffer supplemented with 0.01 mM CaCl_2_, 0.01 mM MnCl_2_, and 0.01 mM MgCl_2_ for 2 hours at room temperature. Sections were then washed 3 × 10 min with 0.1 M PBS before exchanging with mounting medium (VECTASHIELD Mounting Medium with DAPI, H-1200) and placing coverslips.

### Detection task in mice

To determine their perceptual thresholds, we used a two-alternative forced choice task to train seven mice in a vibrotactile detection task (*29*). Mice were trained to lick, in the response period, toward either a right or left reward spout if a vibrotactile stimulus was present or absent during the preceding stimulus period, respectively. All other experimental conditions were as described in the previous study (*29*). Correct responses were rewarded with a drop of water at the corresponding spout, and incorrect responses were punished by a timeout. Trials without a response were neither rewarded nor punished and occurred on <5% of trials. To minimize a direction bias, the trial type was chosen pseudo-randomly by allowing a maximum of three trials of the same type in a row (50% chance of occurrence for each otherwise). We tested the perceptual thresholds at seven different frequencies (300, 450, 700, 1000, 1300, 1600, and 2000 Hz) in separate sessions and in randomized order. Between one and three sessions were tested in a single day, and the same session (i.e., frequency) was repeated up to five times on separate days per mouse. Before testing, the mice were first trained on all frequencies at the largest possible amplitude that the actuator could produce at each frequency. This value ranged from 10 μm (at 300 Hz) to 1 μm (at 2000 Hz). The training lasted 12 to 24 days. Testing of each frequency started at the largest possible amplitude and was progressively attenuated in −4-dB steps after every six vibration trials (total of ≈12 trials) if the proportion of correct responses exceeded 70%. The amplitude was increased by 4 dB if the proportion of correct responses decreased below 60% after every ≈12 trials (including at least six vibration trials). To determine the perceptual threshold at each frequency, we compared the ratio of correct responses for each bout of trials at a given amplitude to chance (i.e., 0.5) using the one-sided binomial test. The threshold was the lowest amplitude of the session for which the test yielded a significance level of <0.05. The thresholds of repeated sessions were averaged and allowed establishing the V-shaped vibrotactile sensitivity curves.

### Data analysis

#### Significant responses

For electrophysiological recording, tuning curves were computed by the spike rate of the neurons. To ensure that we were recording from a single nerve fiber/neuron and to avoid the complications of spike sorting, we only sampled units with a signal-to-noise ratio (SNR) > 5, when responding to a vibration stimulation (100 or 700 Hz). SNRs were calculated as the maximum amplitude of the mean spike waveform divided by the SD of the background noise. There was no additional offline filtering performed for spike detection.

#### Tuning curve fitting

Neuronal structures were considered to be responsive if the maximum stimulus-related spiking responses to any stimulus was greater than 5% on average, and also greater than 2 SDs above the mean firing rate. In addition, we required that neurons responded at least 2 SDs above baseline on at least 20% of the trials tested. The same criteria were used to identify neurons with significant responses to the vibration, optogenetic, and stimuli. Neuronal structures were considered to be frequency selective if they were responsive and also met the following criteria: (i) well fit by the polynomial function (*r* > 0.7, *P* < 0.05) and (ii) tuning index (TI) > 0.2

$$\text{TI}=\frac{\mu_{\text{pref}}-\mu_{\text{ortho}}}{\mu_{\text{pref}}+\mu_{\text{ortho}}}$$

where μ_pref_ denotes the mean response to the preferred stimulus frequency and μ_ortho_ is the mean response to the least preferred stimulus frequency, defined by the curve fitting. To characterize a neuron’s tuning to vibration frequency, the normalized mean responses were fit to frequency by the polynomial curve fitting function (Matlab) using the method of nonlinear least squares, with the degree of polynomial fit set at 6. The tuning preference of individual neurons was defined by the peak of the curve, and the tuning width was defined by the half width at half maximum of the curve.

#### Phase-locked spiking

To determine whether a nerve fiber or a neuron was entrained by the sinusoidal vibration, we calculated standardized inter-spike intervals (SISIs). We chose this measure because it is immune to variability in response onset times across different stimulus repetitions. For a given vibration frequency, the inter-spike intervals (ISIs) of all possible spike pairs that occurred during stimulation were calculated and grouped across stimulus repetitions. Because entrained spiking should yield an ISI distribution that peaks at integer multiples of the sinusoidal stimulus period *T*, values were converted to SISIs according to SISI = *T* + (ISI − *nT*), where *nT* is the integer multiple of *T* closest to ISI. Entrainment probability was defined as the percentage of ISIs in the [*nT* − *T*/12, *nT* + *T*/12] interval. Given that standardized ISIs are distributed between *nT* − *T*/2 and *nT* + *T*/2, entrainment probability should equal unity in the case of perfect entrainment and be equal to one-sixth in the case of chance entrainment. For each neuron and at each frequency, we repeated the calculation of entrainment probability 1999 times with randomly sampled ISIs (with replacement). We then measured whether the lower 99th percentile of the repeated measures was less than one-sixth, which constitutes a one-tailed bootstrap test at significance level *P* < 0.01.

#### Quantification of surface areas of individual LSCs and the axon

To quantify the surface areas of individual LSCs and the axon, all annotations were first downloaded through WebKnossos as TIFF stacks. These were followed by the extraction of individual segmentations using Fiji/ImageJ, based on their “Segment ID” labeled in WebKnossos, separately. After setting the pixel-to-micrometer scale, the “Analyze Particles” function of Fiji/ImageJ was applied to process all electron microscopy (EM) images and export the perimeter for the selected segment (Schwann cell or the axon). The surface area for that selected segment was calculated by multiplying the total perimeter by the *z*-step size of 0.05 μm [surface area (μm^2^) = total perimeter (μm) × 0.05 (μm)]. To quantify the surface areas in different quadrants (transverse view) or along the longitudinal direction, the annotations were cropped or selected before processing with the Analyze Particles function.

#### Quantification of LSC-axon and LSC-LSC contacts

LSC-axon contacts occur at the axon protrusion or body. Each interaction between the LSCs and the axon protrusion is characterized by tiny, sparse contacts, while LSCs contact the axon body in a patchy pattern. We manually determined the first contact position, number of contacts (distance <30 nm), and contact range between individual LSCs and the axon protrusion in all EM images. The estimated size of each contact is ~0.1 μm. The total contact area with the axon protrusion was calculated by summing the contact range (μm) and multiplying by 0.1 μm. To determine the total contact area of an individual Schwann cell with the axon (protrusion and body), we used the “MorphoLibJ” plugin in Fiji/ImageJ. We performed morphological dilation to expand the axon boundaries by 30 nm, measured the overlap perimeter between the dilated axon and LSCs, and then multiplied half of this perimeter by the *z*-step size of 0.05 μm. LSC-LSC contacts were measured similarly to LSC-axon contacts. To determine the contact area of one LSC with other LSCs, we used the MorphoLibJ plugin in Fiji/ImageJ. We performed morphological dilation to expand the LSC boundaries by 30 nm, measured the overlap perimeter between the dilated LSC and other LSCs, and then multiplied half of this perimeter by the *z*-step size of 0.05 μm.

#### Correlation analysis

The Pearson correlation coefficients (*r*) used in this paper to compare two groups were calculated using GraphPad Prism software. The *P* value of the correlation coefficient is used to determine the significance of the correlation analysis.

### Statistical analysis

Statistical analyses were conducted using GraphPad Prism or Matlab, with *P < 0.05 set as the significance threshold. Detailed descriptions of the statistical tests and sample sizes for each experiment are provided in the figure legends and main text. No statistical methods were used to predetermine sample size, and all experimental animals were included in the analysis. The normality assumption was tested with the Kolmogorov-Smirnov test. Nonparametric tests were used when the normality assumption was not met. We used a two-sided nonparametric Wilcoxon rank sum test or Mann-Whitney test to compare two groups and the Kruskal-Wallis test to compare multiple groups with post hoc tests using Dunn’s test, without assumptions of normality or equal variances. Paired-sample *t* test was used to compare the effect of optogenetic manipulations. All statistical methods were two-sided.
