# Supplementary material for: Lamellar Schwann cells in the Pacinian corpuscle potentiate vibration perception
Source: Sci Adv. 2025 Jun 11;11(24):eadt5110. doi: 10.1126/sciadv.adt5110 (PMC12154225; doi:10.1126/sciadv.adt5110)
Supplement: Supplementary file 1 — Figs. S1 to S10 Table S1 Legend for data S1 [file sciadv.adt5110_sm.pdf]

Science Advances
AAAS

Supplementary Materials for

Lamellar Schwann cells in the Pacinian corpuscle potentiate vibration perception

Yuh-Tarng Chen *et al.*

Corresponding author: Kuo-Sheng Lee, leeku@ibms.sinica.edu.tw; Daniel Huber, daniel.huber@unige.ch

Sci. Adv. **11**, eadt5110 (2025)
DOI: 10.1126/sciadv.adt5110

The PDF file includes:

Figs. S1 to S10
Table S1
Legend for data S1

Other Supplementary Material for this manuscript includes the following:

Data S1

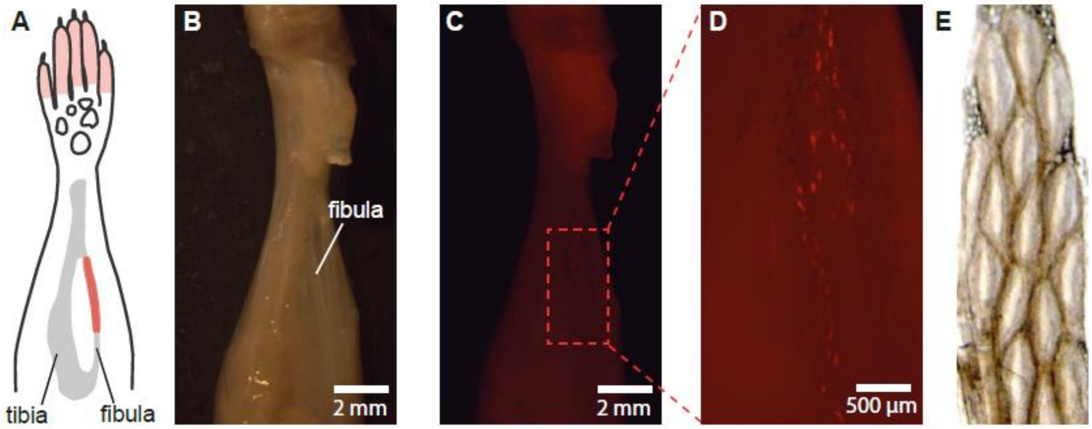

Fig. S1: PCs can be reliably identified and dissected from the fibula of the mouse hindlimb.

(A) Distribution of PCs in the mouse hindlimb.

(B) Mouse hindlimb with muscles removed to exposed tibia and fibula.

(C-D) Dissected hindlimb under a TxRed filter, showing the Etv1+ inner core regions of the PCs (each red fluorescent spot is one inner core).

(E) A bundle of dissected PCs prepared for SBF-SEM, just before resin embedding.

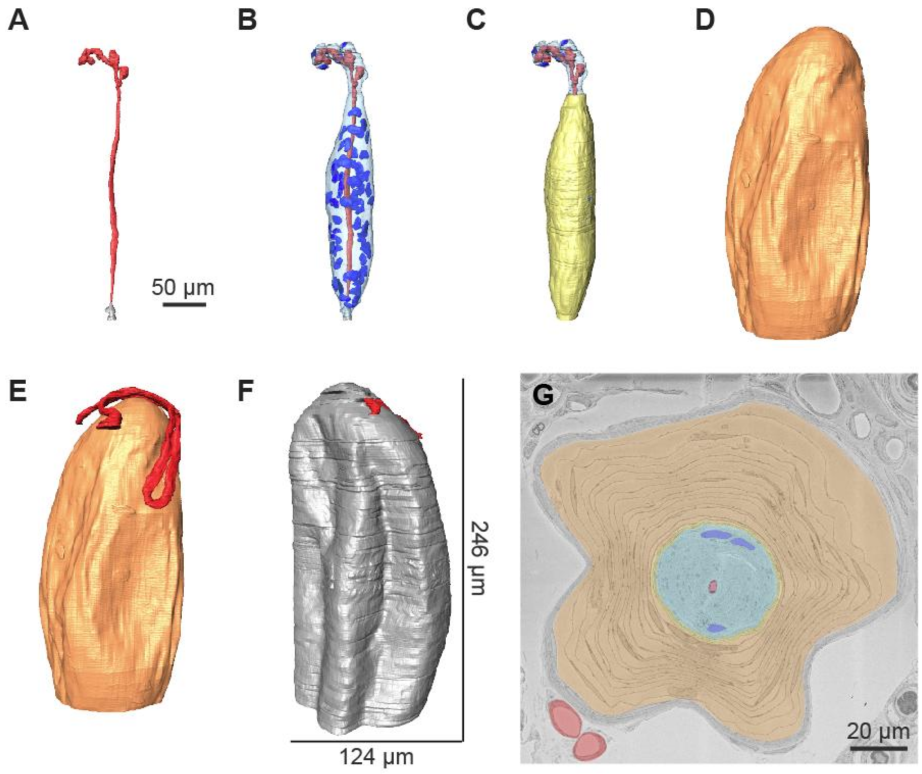

Fig. S2: Reconstructions of SBF-SEM data reveal PC structure in 3D.

(A-F) Manual reconstruction of a full mouse PC, imaged at lower resolution (40 x 40 x 200 nm). The scale bar in B is true for B-G.

(A) The axon (red) is myelinated (gray) in the pre-terminal, long and oval-shaped in the terminal, and finally bulbing and bifurcating in the ultraterminal.

(B) The outline of the inner core (transparent blue) region. At this resolution, the nuclei (opaque blue) in the IC could be reconstructed, but not the layers of the LSCs. In this inner core there were 63 nuclei, suggesting it is made up of as many LSCs.

(C) The intermediate layer (yellow) is uniform in the pre-terminal and terminal, but merged with the outer core layers in the ultraterminal, so that portion could not be reconstructed.

(D) One of fourteen outer core layers (orange).

(E) The blood vessel (red) between the outer core and capsule.

(F) The capsule (gray). At its largest, the PC was 246 µm long and 124 µm wide.

(G) Pseudo-coloured image from the SBF-SEM stack, showing the different reconstructed areas.

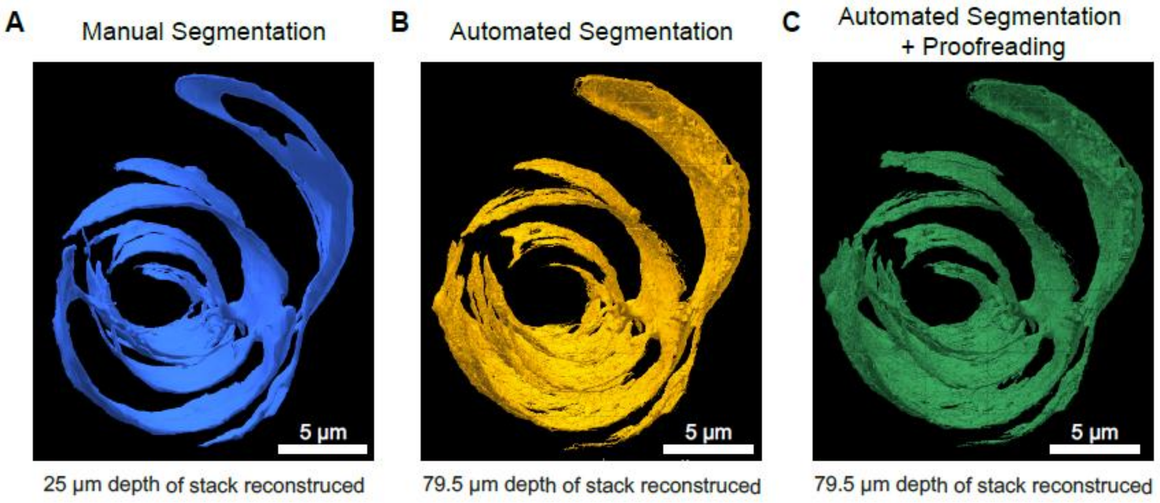

Fig. S3: LSCs segmented by automated methods are equivalent to those segmented manually.

(A) LSC4 when segmented by hand using AMIRA. The segmentation required over 300 hours.

(B) LSC4 when segmented using the Webknossos automated segmentation services.

(C) LSC4 when segmented using the Webknossos automated segmentation services, followed by manual proofreading. The manual proofreading required around 10 hours.

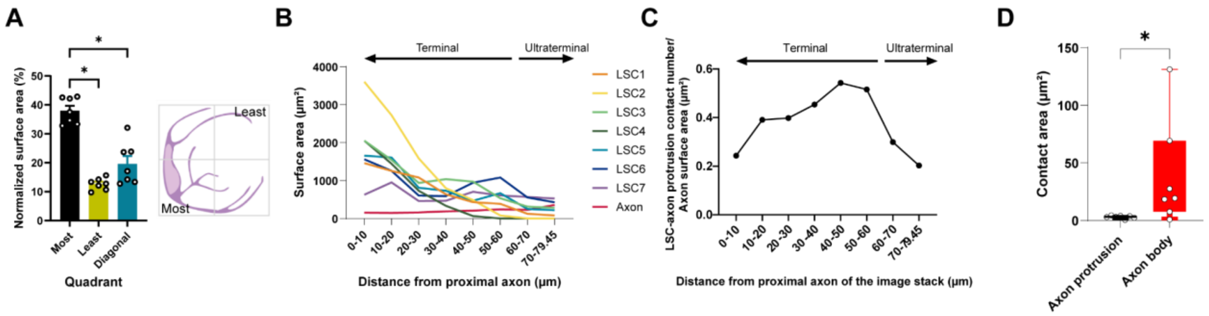

Fig. S4: The distribution of seven lamellar Schwann cell surface areas and their axon protrusion contacts.

(A) The surface area within the quadrant with the most surface area is more than the surface area within its diagonal quadrant and the quadrant with the least surface area. Statistical significance is calculated using T-Tests. *p < 0.05. n= 7.

(B) The lamellar Schwann cells surface area decreases proximally to distally (statistical significance is calculated using One-way ANOVA. (F(7,48) = 11.88, *P < 0.0001, n = 7), while the axon surface area increases.

(C) The density of LSC-axon protrusion contacts from proximal to distal.

(D) LSCs make more contacts with the axon body than with the axon protrusion. Statistical significance is calculated using T-Tests. *p < 0.05. n= 7.

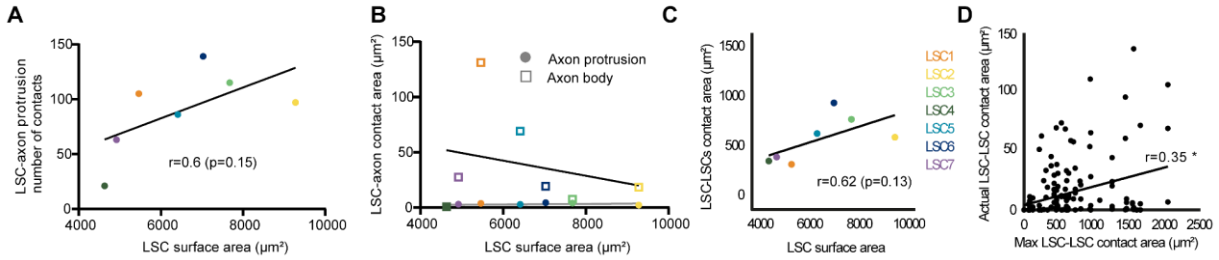

Fig. S5. A larger LSC surface area might lead to more axon protrusion contacts but not necessarily to more axon contact area.

(A) There is a trend of positive correlation between the size of the surface area and the number of axon protrusion contacts.

(B) There is no apparent correlation between the size of the surface area and the size of the LSC-axon contact area.

(C) LSC surface area appears to be positively correlated with the size of the LSC-LSC contact area.

(D) The bigger anatomical presence of two LSCs among the same region can predict the larger LSC-LSC contact area (see **Methods** for details). The relationship between the theoretical maximum LSC-LSC contact area and the measured LSC-LSC contact area is statistically significant.

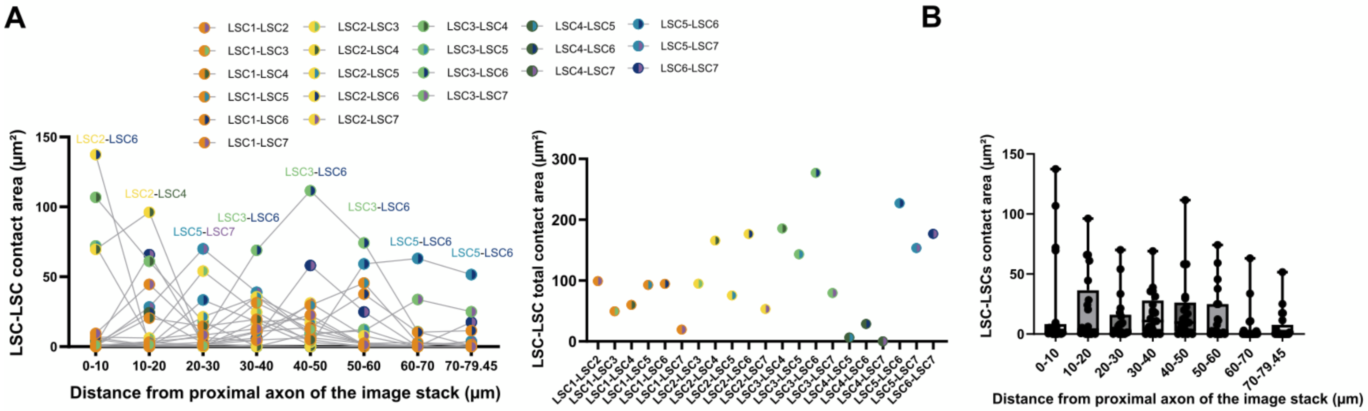

Fig. S6. The distribution of LSC-LSC contacts in the PC inner core.

(A) LSC-LSC contact area measured at different distances from the proximal axon of the image stack, along with their overall contact with the axon.

(B) The Box plot shows the distribution of LSC-LSC contact areas across the same distance intervals as in **(A)**, and there is no significant difference from proximal to distal (One-way ANOVA; F (7, 160) = 1.279).

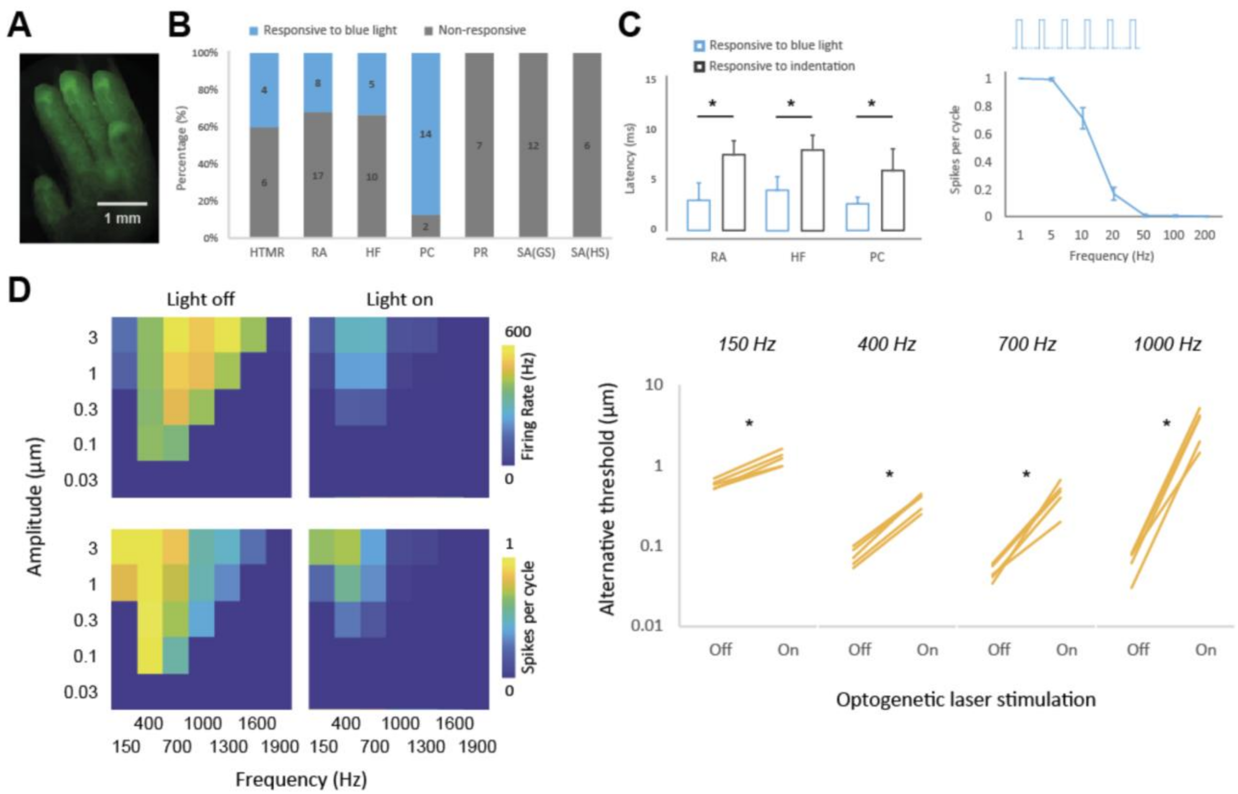

Fig. S7. Characterizing the mechanoreceptor types in Etv1-ChR2 mice.

(A) The EYFP signal within the hindpaw digit area.

(B) Total number of mechanoreceptors recorded in Etv1-ChR2 mice, showing proportions of light responsive (blue) and unresponsive mechanoreceptors (gray). HTMR, high-threshold mechanoreceptor; RA, rapidly-adapting receptor; HF, hair follicle; PC, Pacinian corpuscle; PR, proprioceptor; SA, slowly-adapting receptor; GS, glabrous skin; HS, hairy skin.

(C) Left, first spike latencies from optogenetic activation of Schwann cells and mechanical activation of the same afferent by an indentation (unpaired t-test, *P<0.05). Right, 1-1 spiking can only be reliably activated by optogenetic stimulation at the low frequency below 5 Hz (5mW, 1ms laser pulses).

(D) Force-frequency tuning curves from 5 neurons (Left). Alternative threshold defined with significant entrainment (Right, see **Methods**).

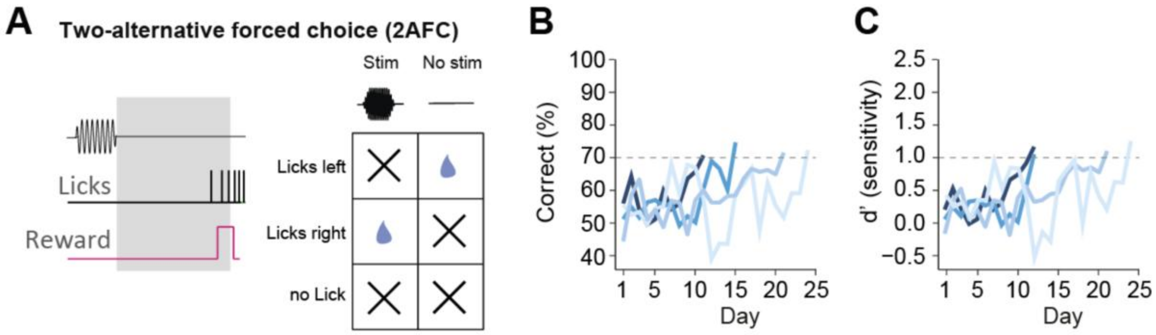

Fig. S8. Behavioral training for vibration detection tasks.

(A) Structure of the 2AFC behavioral task. During stimulus trials, mice licking right following stimulus onset were rewarded and classified as hits. During no stimulus trials, licks right were recorded as miss. d' were calculated to assess performance.

(B) Mice were trained to report the presence of subtle vibrotactile stimuli by licking right and no stimulation by licking left. The correction rate of 5 mice showed a gradual increase over the course of training.

(C) Mice that reached a performance level of d' > 1 were considered as successfully trained.

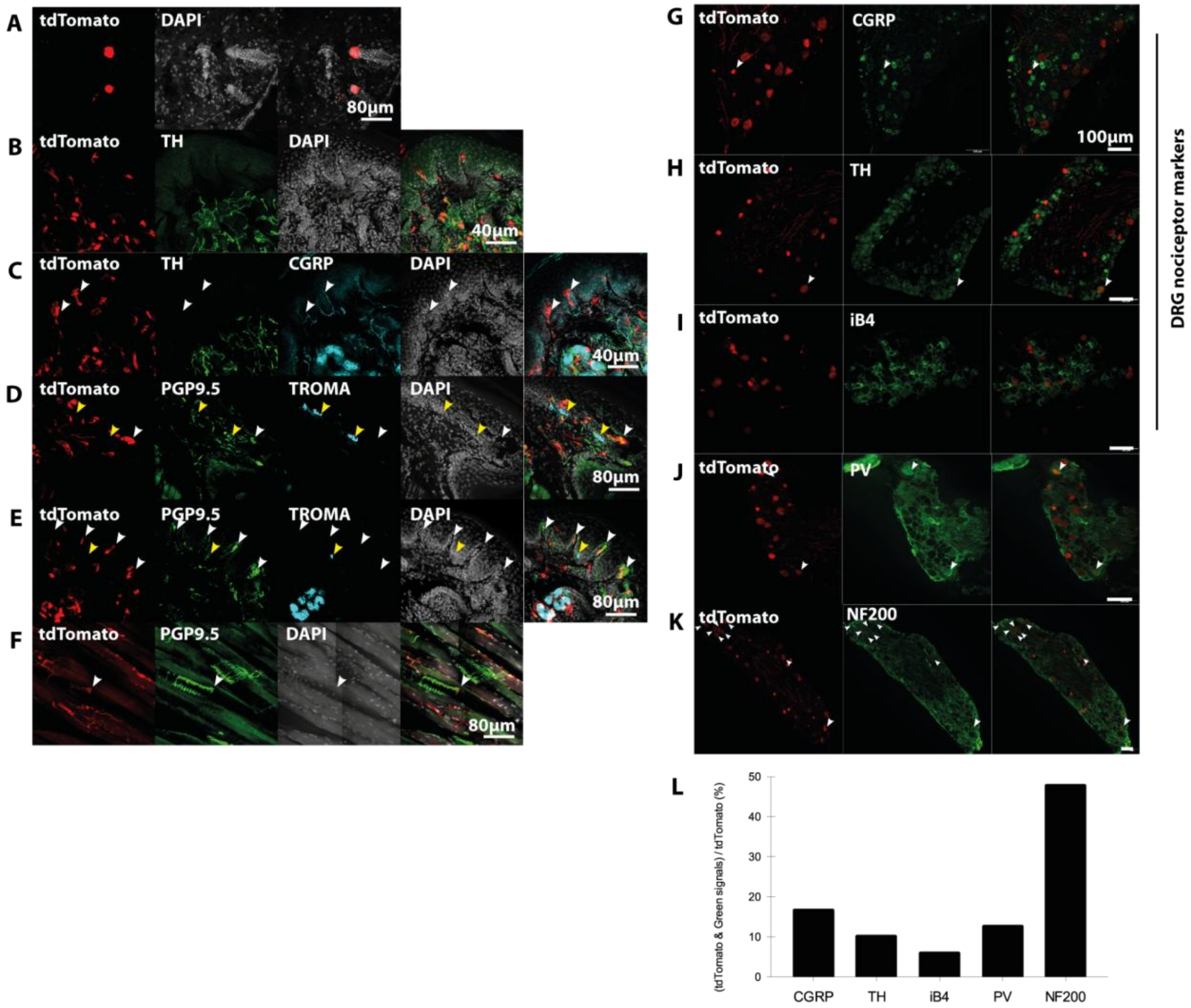

Fig. S9. Etv1 expression in peripheral afferents and receptors.

(A) Etv1-tdTomato expression in sebaceous glands of hair cells, but not in hair cell afferents.

(B) Etv1-tdTomato expression associates with TH fibres near vasculature below the subdermis, but not in the skin.

(C) Etv1-tdTomato expression in LSCs of Meissner corpuscles, which are also innervated by terminal endings of CGRP fibres. White arrows indicate CGRP+ afferents closely associated with Etv1+ Schwann cells in Meissner corpuscles.

(D-E) Etv1-tdTomato expression in LSCs of Meissner corpuscles, but doesn't appear to be associated with Merkel's cell-neurite complexes, labeled by TROMA-1. White arrows indicate Etv1+ Schwann cells in Meissner corpuscles. Yellow arrows indicate TROMA-1+ Merkel cells.

(F) Etv1-tdTomato expression visible in vasculature and pericytes of muscle tissues, but appear absent, or very weak expression, in muscle spindles (PGP9.5). White arrow indicates a weak Etv1 signal inside a muscle spindle, which may be a blood vessel, like in the surrounding tissue.

(G-I) Coexpression proportion of Etv1-tdTomato and nociceptor markers, CGRP, TH, and iB4, in DRG is relatively low. (J) Coexpression proportion of Etv1-tdTomato and PV in DRG is relatively low.

(K) Etv1-tdTomato expression in DRG, some of which are also expressed NF200.

(L) Percentage of co-localized cells among the Etv1+ DRG neurons, which indicates the composition of Etv1+ DRG neurons not only are largely NF200 positive, but also represent small proportions of various nociceptive populations.

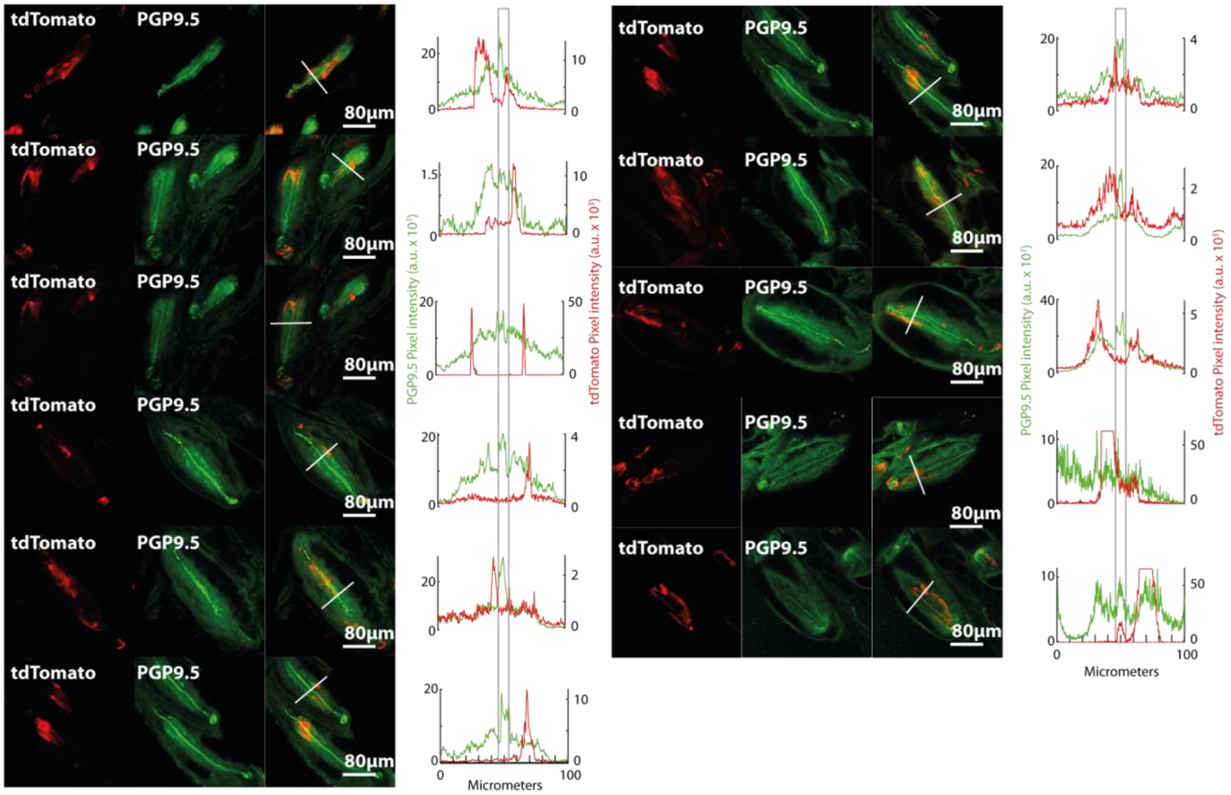

Fig. S10. Characterizing the expression patterns in Etv1-Tdt mice.

Example images of Pacinian corpuscle slices from Etv1-Ai14 (tdTomato) mice, showing tdTomato (Etv1) signal within PCs. PGP9.5 is preferentially expressed in the axon, for comparison. There is strong tdTomato expression in the inner core LSCs, but only in one case (right column, bottom row) did we find a measurable expression of tdTomato in the axon compared to the surrounding LSCs. Quantification of fluorescence intensity of each PC is shown in graphs to the right. Fluorescence intensity was calculated in Fiji over a 100 µm transverse line (white line) through the corresponding PC to the left of the axes. Centred grey box indicates the approximate location of the axon.

Supplementary Table 1. Summary of the structural characteristics of seven Schwann cells.

Schwann cell ID.	Nucleus position* (μm)	First SC-Axon contact position* (μm)	Surface area (μm^2)	Number of SC-Axon protrusion contacts	SC-Axon protrusion contact area (μm^2)	SC-Axon body contact area (μm^2)	Total SC-Axon contact area (μm^2)	SC-SCs contact area (μm^2)
SC1	?-0.05	0.05	5463.2	105	3.76	131.18	134.94	415.81
SC2	?-0.235	2.8	9279.6	97	2.26	18.49	20.75	665.24
SC3	?-0.44	5.85	7676.14	115	4.07	7.56	11.63	830.15
SC4	?-0.745	1.2	4627.2	21	0.38	0.87	1.25	446.6
SC5	?-12.2	24.35	6412.58	86	2.91	69.19	72.1	698.9
SC6	2.6-16.75	29.8	7028.18	139	4.42	19.25	23.67	980.92
SC7	12-20.45	34.6	4919.32	63	3	27.59	30.59	482.81

* Distance from the axon's proximal side within the image stack

Data S1. Source data of the figures within this study.
